# Adenosine Deaminase-Mediated Purine Dysfunction Leads to DNA Repair Inhibition and Senescence in Sporadic Amyotrophic Lateral Sclerosis

**DOI:** 10.3390/ijms27188236

**Published:** 2026-09-16

**Authors:** Benjamin Hall, Yasmina M. Ebrahim, Joanne L. Sharpe, Sangeet Makhija, Brittany C. S. Ellis, Kari E. Wong, Heather Walker, Miriam Yagüe-Capilla, Hannah O. Timmons, Arian Bradley, Ella Nightingale, Rees Ross, Chloe F. Allen, Noemi Gatto, Herbie Garland, Nikita Soni, Stephen J. Kolb, J. Robin Highley, Guillaume M. Hautbergue, Sean G. Rudd, Ryan J. H. West, Pamela J. Shaw, Scott P. Allen

**Affiliations:** 1 Sheffield Institute for Translational Neuroscience, University of Sheffield, Sheffield S10 2HQ, UK; benjamin.hall@sheffield.ac.uk (B.H.); yasmina.maher@pharma.cu.edu.eg (Y.M.E.); joanne.sharpe@sheffield.ac.uk (J.L.S.); s.makhija@sheffield.ac.uk (S.M.); b.ellis@sheffield.ac.uk (B.C.S.E.); htimmons1@sheffield.ac.uk (H.O.T.); ella.nightingale@kcl.ac.uk (E.N.); rees_ross@hotmail.com (R.R.); chloeallen_92@hotmail.com (C.F.A.); noemigatto11@gmail.com (N.G.); herbiegarland2001@gmail.com (H.G.); nsoni3@sheffield.ac.uk (N.S.); robin.highley@sheffield.ac.uk (J.R.H.); g.hautbergue@sheffield.ac.uk (G.M.H.); r.j.west@sheffield.ac.uk (R.J.H.W.); pamela.shaw@sheffield.ac.uk (P.J.S.); 2 Metabolon Inc., Morrisville, NC 27560, USA; wong1524@gmail.com; 3 biOMICS Mass Spectrometry Facility, University of Sheffield, Alfred Denny Building, Western Bank, Sheffield S10 2TN, UK; h.j.walker@sheffield.ac.uk; 4 SciLifeLab, Department of Oncology-Pathology, Karolinska Institute, 41012 Stockholm, Sweden; myague-ibis@us.es (M.Y.-C.); sean.rudd@scilifelab.se (S.G.R.); 5 Instituto de Biomedicina de Sevilla (IBiS), Avda Manuel Siurot S/N, 41013 Seville, Spain; 6 Departamento de Biologia Celular, Facultad de Biologia, Universidad de Sevilla, Avenida Reina Mercedes 6, 41012 Seville, Spain; 7 Department of Neurology, The Ohio State University Wexner Medical Center, 410 W. 10th Avenue, Columbus, OH 43210, USA; stephen.kolb@osumc.edu; 8 Department of Biological Chemistry & Pharmacology, The Ohio State University Wexner Medical Center, 370 W. 9th Avenue, Columbus, OH 43210, USA

**Keywords:** ADA, ALS, astrocyte, metabolomics, MND, purine metabolism

## Abstract

Amyotrophic lateral sclerosis (ALS) is a neurodegenerative disease characterised by the death of motor neurons leading to paralysis and death generally 3–5 years post-symptom onset. ALS is a cell- and non-cell-autonomous disease, with glia such as astrocytes influencing disease pathology and progression. Our laboratory has previously identified purine metabolism dysfunction in induced neural progenitor cell-derived astrocytes (iAstrocytes) from sporadic ALS (SALS) cases, driven by loss of the enzyme adenosine deaminase (ADA). Here, we have demonstrated that loss of ADA, along with changes to ecto-5′-nucleotidase and hypoxanthine-guanine phosphoribosyl transferase led to disruption in purine metabolite levels, linked to the level of the ADA enzyme. These alterations were recapitulated in SALS CSF and post-mortem tissue, with ageing and sex affecting purine metabolite levels downstream of ADA and positively correlating with disease progression. Loss of ADA led to reduced 53BP1-mediated DNA repair and increased P16 levels, which was recapitulated in control iAstrocytes via ADA inhibition. Our findings indicate that TDP43 dysfunction drives impairment of ADA-mediated purine metabolism in vitro, leading to downstream effects that include DNA damage, likely through inhibition of DNA repair mechanisms, and the induction of cellular senescence. Furthermore, these results suggest that therapeutic targeting of the ADA pathway may help slow ALS disease progression.

## 1. Introduction

Amyotrophic lateral sclerosis (ALS) is a neurodegenerative disease that typically develops with age and is caused by the progressive loss of motor neurons in the motor cortex, brainstem and spinal cord. This leads to increasing paralysis and ultimately death, most often due to respiratory failure within 3–5 years after symptoms begin [1]. The global prevalence of ALS is rising, making it an increasingly significant burden on healthcare systems worldwide [2,3,4]. ALS is predominantly sporadic in nature. However, there is crossover with genetic cases, such as in the context of C9orf72-ALS, which accounts for 7–8% of all ALS cases in Western populations, both familial and sporadic [5,6,7,8]. ALS is a non-cell-autonomous disease, with astrocytes conveying neurotoxicity to motor neurons from both familial and sporadic cases. Mechanisms of astrocyte toxicity include, but are not limited to, reduced lactate production, inflammatory release, oxidative stress, extracellular vesicle dysfunction, microRNA dysregulation, connexin dysfunction and purine metabolism disruption [9,10,11,12,13]. We have previously shown that both induced neural progenitor cell (iNPC)-derived C9orf72-iAstrocytes and SALS-iAstrocytes were less able to metabolise the purine nucleoside adenosine, due to a reduction in the enzyme adenosine deaminase (ADA) [10,14]. ADA-mediated purine dysfunction represents an important pathway in ALS, as it influences energy metabolism, antioxidant synthesis, DNA methylation, and the DNA damage response [15,16,17,18,19]. All of these processes have previously been associated with ALS pathology [20]. In a recent publication, we linked ADA loss and purine metabolism dysfunction in C9orf72-ALS to dipeptide repeat protein (DPR) production, with our iAstrocyte data recapitulated in mechanistic models, patient-derived biofluids and in an in vivo *Drosophila melanogaster* C9orf72-DPR model [14,21].

In this linked follow-up study, much of which was performed alongside the C9orf72-iAstrocyte model work, the aim was to establish the downstream functional consequences of ADA loss in SALS-iAstrocytes, establish a cause, and assess whether the in vitro findings could be recapitulated in patient tissue, biofluids and in vivo. Based on our previous work, we hypothesised that SALS-iAstrocytes would show downstream purine metabolism alterations due to loss of ADA. Here, we demonstrate that, along with loss of ADA, further key enzymes involved in the purine metabolism pathway are dysregulated at both the protein and RNA levels and that purine metabolism dysfunction is intrinsically linked to ADA levels, which can influence DNA repair mechanisms and senescence. Furthermore, we show that our ADA-mediated purine metabolism correlates with age at death and disease progression in SALS cases. Finally, our data may suggest a link between ADA-mediated dysfunction and TDP43 pathology, with TDP43 pathology level negatively correlating with ADA level in iAstrocytes and sodium arsenite treatment leading to loss of ADA.

## 2. Results

### 2.1. ADA Loss Leads to Purine Metabolism Alterations in SALS-iAstrocytes

**Figure 1:**
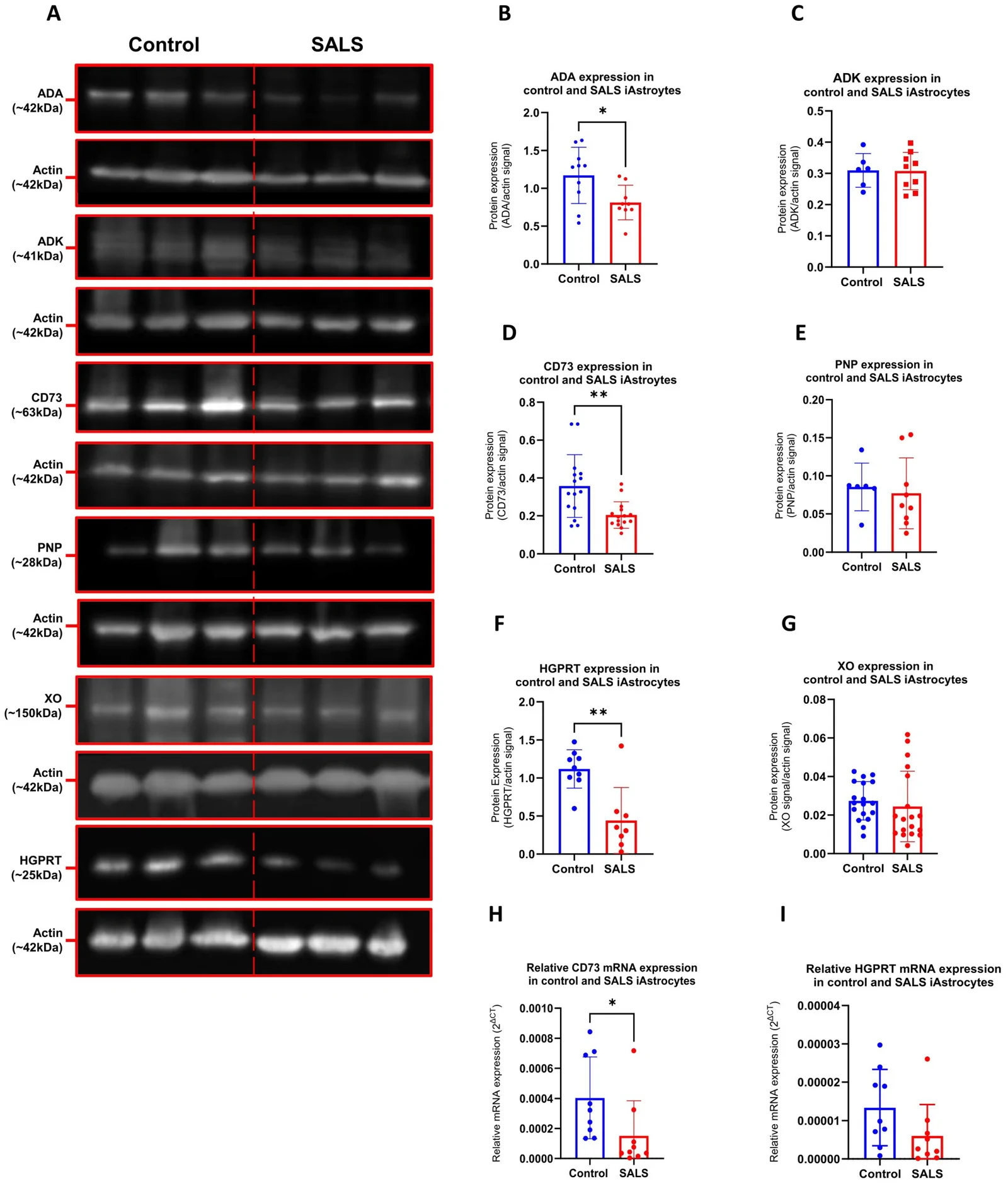
**CD73 and HGPRT levels are reduced in SALS-iAstrocytes.** (**A**) Purine metabolism enzyme Western blot representative images. (**B**) ADA Western blot densitometry analysis. (**C**) ADK Western blot densitometry analysis. (**D**) CD73 Western blot densitometry analysis. (**E**) PNP Western blot densitometry analysis. (**F**) HGPRT Western blot densitometry analysis. (**G**) XO Western blot densitometry analysis. (**H**) CD73 relative mRNA level. (**I**) HGPRT relative mRNA level. Data presented as mean and standard deviation of three biological replicates from three control and three SALS-iAstrocytes. Fold change was calculated relative to a control average from a given assay. Statistical analysis by unpaired *t*-test (**B**,**D**) or Mann–Whitney test (**H**). * *p* ≤ 0.05, ** *p* ≤ 0.01. Where *p* value is not indicated, results were non-significant. Uncropped Western images can be found in Supplementary Materials.

To follow up on our previous work highlighting loss of ADA at the RNA and protein levels in SALS-iAstrocytes [10], we initially performed in-depth characterisation of ADA function and purine metabolism in the same patient-derived cell model. As with our previous C9orf72-iAstrocyte approach, we initially examined the levels of several other enzymes involved in both purine metabolism and salvage in iAstrocytes in addition to ADA, which, as previously observed, was reduced in 2 out of 3 SALS cases (Figure 1A,B). In C9orf72-iAstrocytes, ecto-5′nucleotidase (CD73), which catalyses the dephosphorylation of extracellular adenosine monophosphate (AMP) to adenosine, was reduced, which was also observed in SALS-iAstrocytes at the protein and RNA levels (Figure 1A,D,H). In our previous work, we showed that hypoxanthine guanine phosphoribosyl transferase (HGPRT), which catalyses purine salvage by converting hypoxanthine to inosine monophosphate (IMP), was significantly increased in C9orf72-iAstrocytes compared to controls [14]. In contrast, SALS-iAstrocytes had significantly reduced HGPRT protein levels compared to controls, suggesting less purine salvage (Figure 1A,F). This was somewhat recapitulated at the RNA level without reaching significance (Figure 1I). No other changes in purine enzyme levels were observed, suggesting that purine metabolism from adenosine/deoxyadenosine to urate via inosine/deoxyinosine, and IMP via hypoxanthine, was altered in SALS-iAstrocytes.

In addition to purine metabolism, we assessed the protein levels of the enzymes involved in purine de novo synthesis, namely adenylosuccinate lyase (ADSL); 5-Aminoimidazole-4-carboxamide ribonucleotide Formyltransferase (ATIC); formylglycinamide ribonucleotide amidotransferase (FGAMS); phosphoribosylglycinamide formyltransferase/phosphoribosylglycinamide synthetase/phosphoribosylaminoimidazole synthetase (GART); and phosphoribosylaminoimidazole carboxylase/phosphoribosylaminoimidazole succinocarboxamide synthetase (PAICS), as well as the chaperone protein Hsp90 (Supplementary Figure S1). PAICS has recently been shown to be reduced in human post-mortem cerebellar tissue and iPSC-derived C9orf72 and sporadic ALS motor neurons [22]. Moreover, in our recent C9orf72 work, we showed that although PAICS was not reduced at the protein level, PAICS-mediated purinosome formation was reduced in C9orf72-iAstrocytes [14]. In our SALS-iAstrocyte cohort, PAICS levels were unchanged, as were all other targets assessed aside from ATIC, which was reduced (Supplementary Figure S1D).

**Figure 2:**
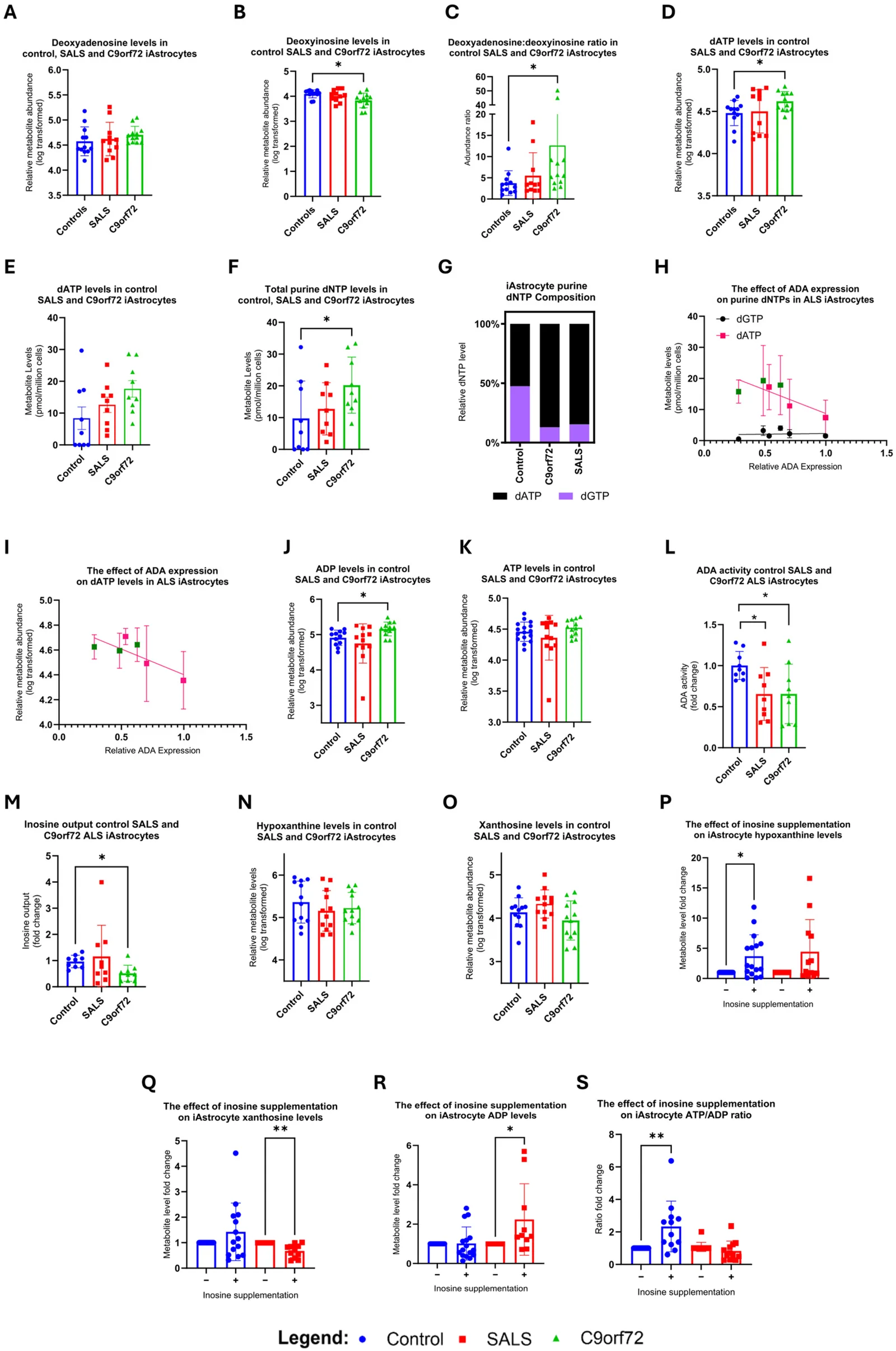
**ADA levels influence purine metabolite levels in iAstrocytes.** (**A**) Deoxyadenosine level. (**B**) Deoxyinosine level. (**C**) Deoxyadenosine: deoxyinosine ratio and (**D**) dATP level as measured via LC/MS. (**E**) dATP level. (**F**) Total purine dNTP level (**G**) and relative purine dNTP composition as measured via a coupled click chemistry and DNA polymerase-based assay. (**H**) Effect of ADA level on purine dNTP output in SALS and C9orf72-iAstrocytes as measured via a coupled click chemistry and DNA polymerase-based assay. Green squares = C9orf72 cases. (**I**) Effect of ADA level on dNTP output in SALS and C9orf72-iAstrocytes as measured via LC/MS. Green squares = C9orf72 cases (**J**) ADP level. (**K**) ATP level. (**L**) ADA activity. (**M**) Inosine output. (**N**) Hypoxanthine level. (**O**) Xanthosine level. (**P**) Hypoxanthine level −/+ Inosine. (**Q**) Xanthosine level −/+ Inosine. (**R**) ADP level −/+ Inosine. (**S**) ATP:ADP ratio −/+/inosine. ADA activity and inosine output were calculated relative to control average from a given assay. iAstrocyte metabolite levels in the presence of inosine were normalised to metabolite levels in the absence of inosine by fold change analysis. Data presented as mean and standard deviation of three or four biological replicates from three controls, three SALS and three C9orf72-iAstrocytes. Statistical analysis performed using Mann–Whitney/unpaired *t*-test (**A**–**O**), linear regression (**H**,**I**) or Wilcoxon test (**P**–**S**). * *p* ≤ 0.05, ** *p* ≤ 0.01. Where *p* value is not indicated, results were non-significant.

We then set out to test our hypothesis that ADA level changes would lead to purine metabolism alterations. We utilised LC-MS-based metabolomics and a deoxyribonucleoside triphosphate (dNTP) measurement assay together with enzyme activity assays to assess whether the loss of CD73, ADA and HGPRT had a functional consequence on purine metabolism in SALS-iAstrocytes and to characterise this pathway (Figure 2 and Figure S2). As previously published [14], the C9orf72 hexanucleotide repeat expansion (C9orf72-HRE) had measurable consequences on purine metabolism in iAstrocytes, including a loss of deoxyinosine, a rise in the deoxyadenosine: deoxyinosine ratio, and an increase in both dATP levels and total purine dNTP levels (Figure 2B–G). In all aspects of the pathway, SALS-iAstrocytes sat between controls and C9orf72-iAstrocytes in terms of severity of effect. This was due to the relative level of ADA within the sporadic subgroup. SALS-iAstrocytes, on average, had higher levels of ADA compared to C9orf72-iAstrocytes, which correlated with levels of purine metabolites, exemplified by correlating ADA levels with dATP and total dNTP levels (Figure 2H,I). Total dNTP and dATP levels negatively correlated with ADA levels in C9orf72 and SALS (green and pink squares respectively, total dNTP r = −0.769, *p* = 0.074, dATP r = −0.780, *p* = 0.059). These data suggest that the purine metabolism defects observed in this study and the preceding study [14] were due to the level of ADA; the higher the level of enzyme, the less severe the phenotype.

This theory was further supported by ADA activity assays and inosine output production measurements (Figure 2L,M), which showed a similar phenotype, providing orthogonal validation to our Western and LC-MS approaches. Interestingly, some subtle SALS-iAstrocyte-specific effects were observed in ADP and xanthosine levels (Figure 2J,O), and although inosine supplementation led to an increase in hypoxanthine in SALS-iAstrocytes (as observed in controls and previously in C9orf72-iAstrocytes, Figure 2P), inosine supplementation led to a decrease in xanthosine and an increase in ADP in SALS-iAstrocytes (Figure 2Q,R). Furthermore, inosine led to a drop in ADP in controls and C9orf72-iAstrocytes, leading to an increase in the ATP/ADP ratio (Figure 2S and [14]), which was not observed in SALS-iAstrocytes. The increase in ADP levels with inosine supplementation in the SALS-iAstrocytes suggests a further ATP synthase defect. The increase in Ribose 5-Phosphate (R-5-P) with inosine supplementation in SALS-iAstrocytes suggests that carbon was perhaps being diverted to the ribose pathway instead of purine metabolism or purine salvage (Supplementary Figure S2J).

### 2.2. ADA-Mediated Purine Metabolism Alterations Are Evident in CSF and Post-Mortem Tissue from SALS Cases and Correlate with Disease Progression

**Figure 3:**
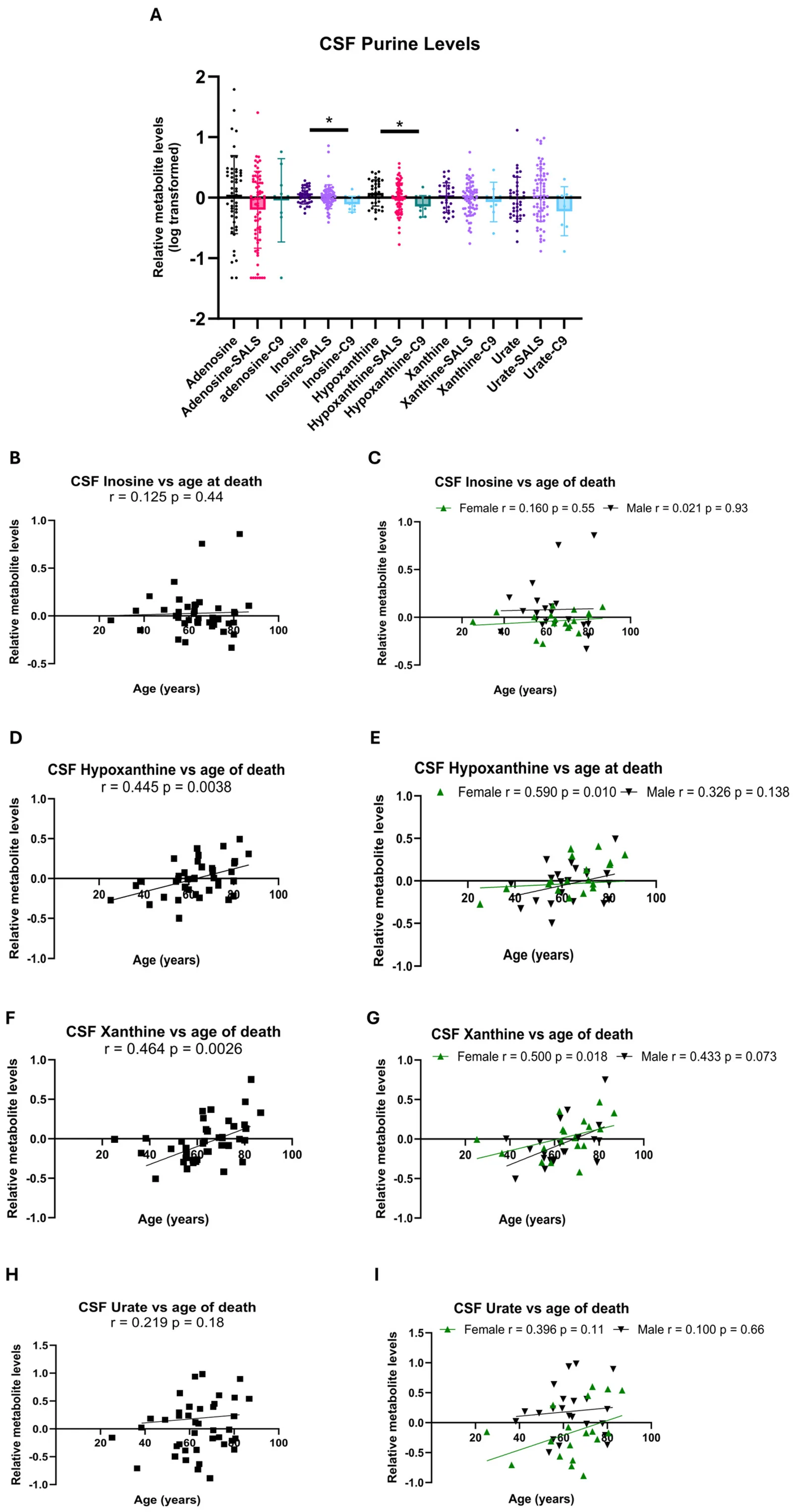
**CSF Purine levels are affected by age and sex in SALS cases**. (**A**) Grouped CSF purine levels. (**B**) Age at death vs. CSF inosine levels. (**C**) Age at death vs. CSF inosine levels in males and females. (**D**) Age at death vs. CSF hypoxanthine levels. (**E**) Age at death vs. CSF hypoxanthine levels in males and females. (**F**) Age at death vs. CSF xanthine levels. (**G**) Age at death vs. CSF xanthine levels in males and females. (**H**) Age at death vs. CSF urate levels. (**I**) Age at death vs. CSF urate levels in males and females. (**A**) Data presented as mean and standard deviation in all CSF cases, with statistical analysis performed by unpaired *t*-test. (**B**–**H**) Data presented for each individual SALS case, and statistical analysis performed by Pearson’s or Spearman’s correlation analysis. * *p* ≤ 0.05. Where *p* value is not indicated, results were non-significant.

To meet our aims and assess whether this ADA level effect on purine metabolism was recapitulated in sporadic patient biofluids, we performed UPLC-MS/MS in the CSF and plasma of a total of 81 SALS cases and age/sex matched healthy controls (Figure 3). At the group level in CSF, the iAstrocyte data were recapitulated, with reduced abundance of purine metabolites downstream of ADA evident in the C9orf72 cases, with sporadic cases showing a less severe phenotype compared to C9orf72 cases (Figure 2A [14]). To assess whether age had an effect on metabolite levels in the CSF, purine levels downstream of ADA function were correlated with age at death in SALS cases. Downstream of the action of ADA, the purine metabolites hypoxanthine and xanthine positively correlated with age at death, a correlation that was lost at the level of urate (Figure 3D,F,H). To assess the effect of sex, the SALS cases were split into males and females, and the ageing analysis was repeated. In females, hypoxanthine and xanthine levels positively correlated with age at death, whereas in males, only a trend was observed (Figure 3E,G). At the level of urate, no significant correlations with age at death in males or females were observed (Figure 3I).

**Figure 4:**
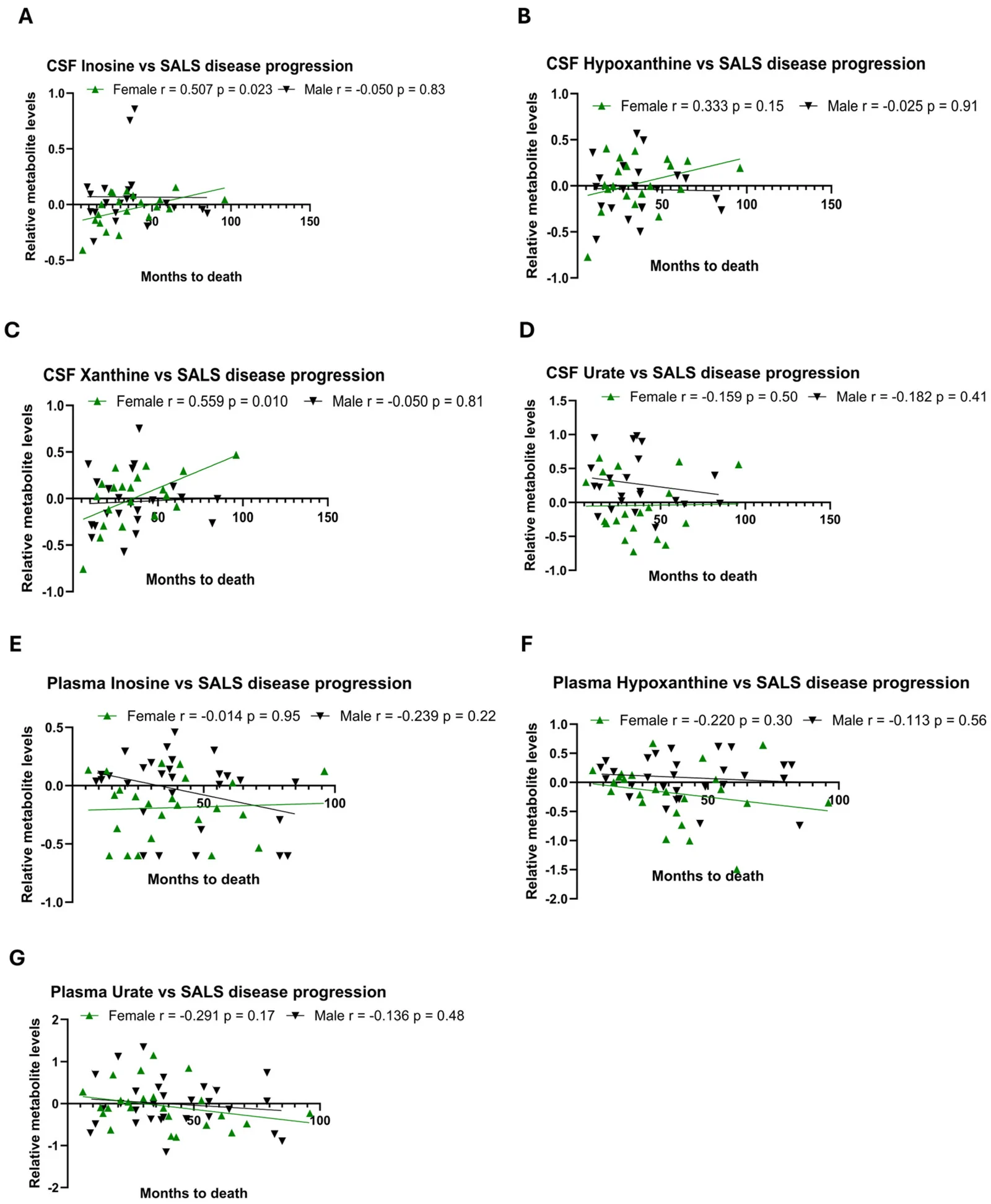
**CSF Purine levels positively correlate with disease progression length in female SALS cases.** (**A**) CSF inosine levels vs. disease progression length in male and female SALS cases. (**B**) CSF hypoxanthine levels vs. disease progression length in male and female SALS cases. (**C**) CSF xanthine levels vs. disease progression length in male and female SALS cases. (**D**) CSF urate levels vs. disease progression length in male and female SALS cases. (**E**) Plasma inosine levels vs. disease progression length in male and female SALS cases. (**F**) Plasma hypoxanthine levels vs. disease progression length in male and female SALS cases. (**G**) Plasma urate levels vs. disease progression length in male and female SALS cases. Data presented for each individual SALS case, and statistical analysis performed using Pearson’s or Spearman’s correlation analysis.

When correlating CSF metabolite levels with age at onset (Supplementary Figure S3), similar trends were observed, but much of the significance was lost, indicating a post-symptom onset change in purine metabolite metabolism. One difference was urate, which showed a near-significant positive correlation with age at onset at the group level that was driven by males in the cohort (Supplementary Figure S3G,H), suggesting a SALS age at onset effect not observed upstream in initial purine metabolism. Performing the same analysis in the CSF of control cases showed that at the group level, a natural ageing effect in hypoxanthine and xanthine was evident (Supplementary Figure S4C–F), which could account for the SALS ageing data, although the SALS data showed a muted response compared to controls. Therefore, to establish a firm link between disease and purine metabolite levels, disease progression in months from symptom onset to death was correlated with purine metabolites downstream of ADA (Figure 4). At the group level, no significant changes with disease progression were observed (n. However, when sex was taken into account, inosine, hypoxanthine and xanthine levels all showed a positive correlation with disease progression in females, not males (Figure 4A–C). This suggests that higher levels of purine metabolites downstream of ADA may be linked with longer disease progression post-symptom-onset in females.

**Figure 5:**
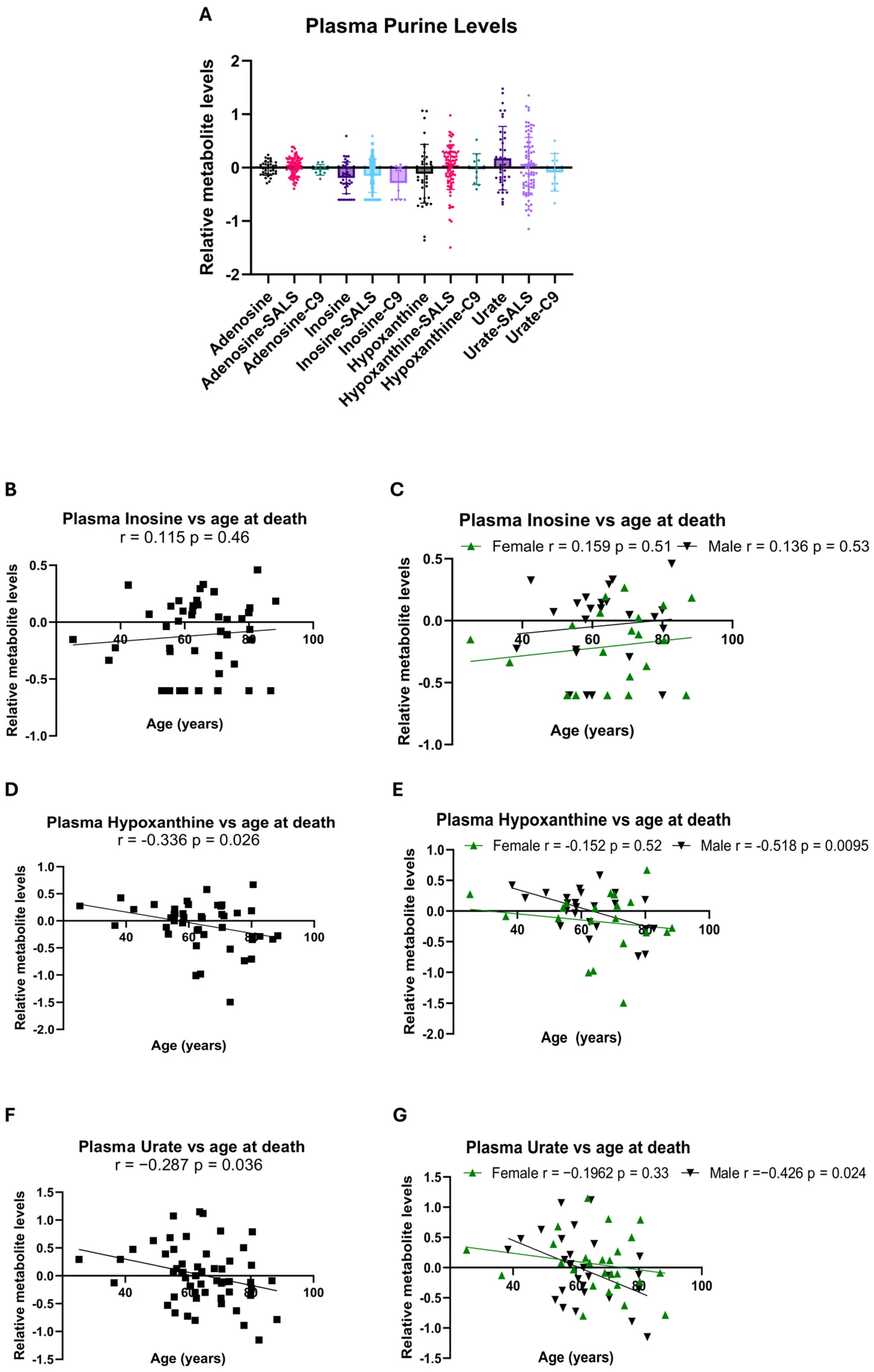
**Plasma purine levels are affected by age and sex in SALS cases**. (**A**) Grouped plasma purine levels. (**B**) Age at death vs. plasma inosine levels. (**C**) Age at death vs. plasma inosine levels in males and females. (**D**) Age at death vs. plasma hypoxanthine levels. (**E**) Age at death vs. plasma hypoxanthine levels in males and females. (**F**) Age at death vs. plasma urate levels. (**G**) Age at death vs. plasma urate levels in males and females. (**A**) Data presented as mean and standard deviation in all CSF cases. (**B**–**G**) Data presented for each individual SALS case, and statistical analysis performed by Pearson’s or Spearman’s correlation analysis. Where *p* value is not indicated, results were non-significant.

As observed previously with the C9orf72 cases in terms of inosine, hypoxanthine (xanthine was not robustly detected) and urate, no plasma group effect was observed in the SALS cases (Figure 5A). However, when taking age into account, the opposite of the results found in the CSF was observed, in that hypoxanthine and urate levels negatively correlated with age at death in SALS cases, a trend driven by males (Figure 5D–G). This was recapitulated when correlating with age at onset (Supplementary Figure S5) but was not observed in controls to the same extent (Supplementary Figure S6), suggesting a SALS-specific ageing effect that occurs pre-symptom-onset, as the negative correlation was not observed with disease progression (Figure 4E–G).

Overall, these data suggest for the first time that higher CSF purine metabolites and lower plasma purine metabolites downstream of ADA are associated with a slower disease progression in SALS. To test this further, we measured ADA levels by Western blot in the post-mortem grey and white matter motor cortex of SALS cases (Supplementary Figure S7). At the group level, no changes in ADA were observed in SALS cases in both grey and white matter (Supplementary Figure S7A–C), with C9orf72 data shown from our previous study as a comparison [14]. As with our LC-MS data, white matter SALS ADA levels sat between controls and C9orf72 cases. However, when correlating ADA levels with age at death and age at onset in white matter, a positive correlation was observed (Supplementary Figure S7E,G). This correlation was not observed in grey matter or with disease progression (Supplementary Figure S7D,F–I). Overall, these data align with what we see in SALS-iAstrocytes and in CSF from SALS cases, suggesting that at the group level, SALS cases sit between controls and C9orf72 cases in terms of ADA levels. However, these levels may be associated with older age at death and/or disease length.

### 2.3. Reduced ADA Levels Are Not Due to Transcription Factor Dysregulation in Sporadic iAstrocytes

In our previous publication, we showed that DPRs caused by C9orf72 hexanucleotide repeat expansion are the primary driver of ADA-mediated purine dysfunction [14]. However, DPRs are not present in sporadic ALS; therefore, we asked what could be the common factor that links purine dysfunction in C9orf72 ALS and sporadic ALS. We initially focussed on transcription factor regulation, as the ADA gene has previously been shown to be a target of the transcription factors p63 and p73, homologs of the p53 tumour suppressor gene [23,24,25]. Inducing TAp73α expression in human osteosarcoma cell lines resulted in elevated ADA levels, accompanied by alterations in purine metabolism, including increased inosine [26], whilst in epidermal keratinocytes, p63 knockdown correlated with a decrease in ADA levels [27]. Loss of the *TP73* gene has been linked to ALS, with mutations found in SALS cohorts [28]. *TP73* gives rise to two different main protein isoforms, TA-p73 and the brain isoform ΔN-p73, which promotes survival via anti-apoptotic mechanisms [29,30]. We assessed protein levels of ΔN-p73 and p63 by Western blot and RNA levels by q-RT-PCR in the SALS-iAstrocytes and did not find conclusive evidence of reduced levels of either target at the protein level (Supplementary Figure S8A–F).

### 2.4. TDP43 Pathology Is Linked to Reduced ADA Levels in Sporadic iAstrocytes

**Figure 6:**
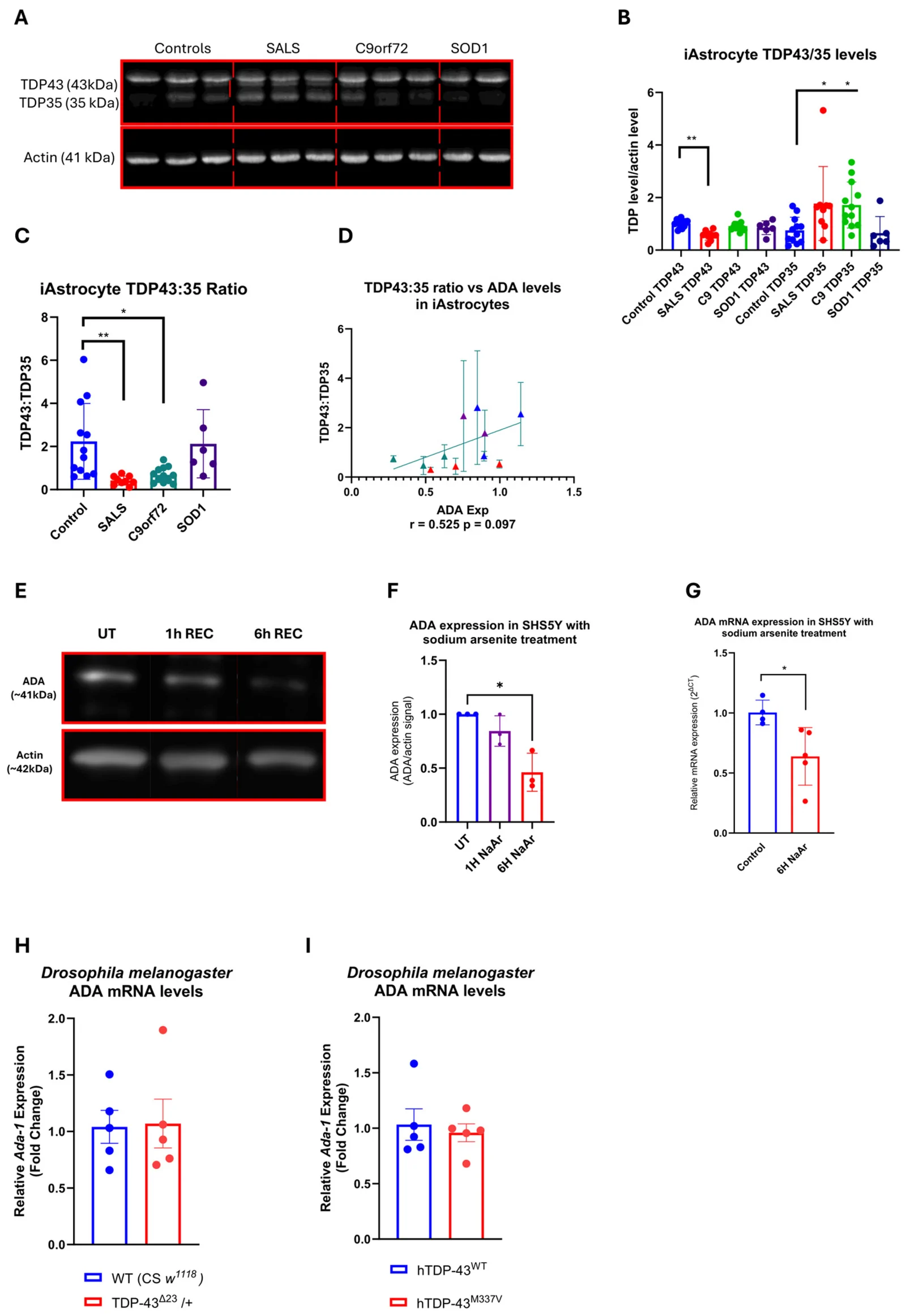
**TDP43 pathology linked to loss of ADA in vitro but not in vivo.** (**A**). Representative TDP43 Western blots in iAstrocytes. (**B**) Densitometry analysis of A. (**C**) Calculated TDP43:35 ratio in iAstrocytes. (**D**) TDP43:35 ratio vs. ADA levels in iAstrocytes. (**E**) Representative ADA Western blots in SY5Ys. (**F**) Densitometry analysis of E. (**G**) ADA mRNA levels in SHY5Y-treated cells. (**H**) ADA mRNA levels in a *Drosophila melanogaster* TDP43∆23 model. (**I**) ADA mRNA levels in a *Drosophila melanogaster* TDP43 M337V model. (**A**–**D**) Data presented as mean and standard deviation of three/four biological replicates from three control, three SALS, three C9orf72- and two SOD1-iAstrocytes. (**E**–**G**) Data presented as mean and standard deviation of three/four biological SHY5Y replicates. (**H**,**I**) Data presented as mean and standard deviation of 5 independent crosses. Analysis performed using Kruskal–Wallis (**B**,**C**), Pearson’s correlation (**D**), Friedman’s test (**F**), Mann–Whitney (**G**) * *p* ≤ 0.05. ** *p* ≤ 0.01. Where *p* value is not indicated, results were non-significant.

TDP43 pathology is observed in up to 97% of all ALS cases, including C9orf72 via DPR production [31], but is predominantly absent in SOD1 and FUS cases. In SALS/C9orf72-ALS, TDP43 is mislocalised from the nucleus, forming cytoplasmic inclusions. Concomitantly, loss of TDP43 from the nucleus leads to RNA regulatory disruption and loss of suppression of cryptic exon splicing [32]. Cytoplasmic TDP43, indicating nuclear mislocalisation, is known to be cleaved, producing C-terminal fragments at 35 kDa [33], which have previously been shown to be elevated in C9orf72 and SALS-iAstrocytes cohorts [34]. We recapitulated these findings, which led to a decrease in the 43 kDa full-length protein, an increase in the TDP35 fragment and a decrease in the TDP43:TDP35 ratio (Figure 6A–C). Two SOD1 patient-derived lines, both with an A4V mutation, did not show evidence of the same level of TDP43 pathology as the C9orf72 and SALS-iAstrocytes and did not show loss of ADA (Supplementary Figure S9). Moreover, ADA levels somewhat correlated with the TDP43:TDP35 ratio in iAstrocytes, suggesting that TDP43 pathology is linked to loss of ADA levels, but this did not reach significance (Figure 6D, *p* = 0.097). To assess this functionally and provide orthogonal validation, we performed sodium arsenite treatment in WT SHS5Y neuronal-like cells, which has been shown previously to lead to TDP43 cytoplasmic aggregation [35]. Six-hour treatment led to a significant loss of ADA at the protein and mRNA levels in SHS5Y cells (Figure 6E–G), without affecting cell viability and the level of GAPDH and U1 (Supplementary Figure S9). Moreover, the level of the purine enzyme PNP was not significantly reduced (Supplementary Figure S9), suggesting an ADA-specific effect, which aligns with our iAstrocyte data. To assess whether these in vitro findings linking TDP43 pathology to ADA levels were recapitulated in vivo, we measured the levels of ADA mRNA in TBPH^Δ23^, TBPH^KO.hTARDBP.WT^ and TBPH^KO.hTARDBP.M337V^ *Drosophila melanogaster* models. We found no difference in ADA levels in either model.

### 2.5. ADA Activity Protects Against P53-Mediated Senescence and DNA Damage in SALS-iAstrocytes

To assess the downstream consequences of loss of ADA function in sporadic ALS, we focussed on the links between purine metabolism, DNA damage and senescence. As part of our transcription factor analysis, we also measured the levels of P53. p53 is a master transcription factor with functions in metabolism, the cell cycle, DNA repair, apoptosis and autophagy [36]. Homeostatic levels of p53 are vital for normal functioning, as are p63 and p73, which share sequence similarities with p53, such as similar DNA-binding domains [37]. P53 at the RNA level was elevated in SALS-iAstrocytes; however, the full-length protein was significantly reduced (Supplementary Figure S8G–I), suggesting an attempted compensatory measure. Interestingly, we noticed an additional band at approximately 47 kDa in the two SALS-iAstrocyte lines with the lowest ADA levels, which ran at the same predicted height as the p53β isoform. p53β has been shown to be a marker of senescence via upregulation of target genes such as p21 in cooperation with full-length p53 [38,39].

**Figure 7:**
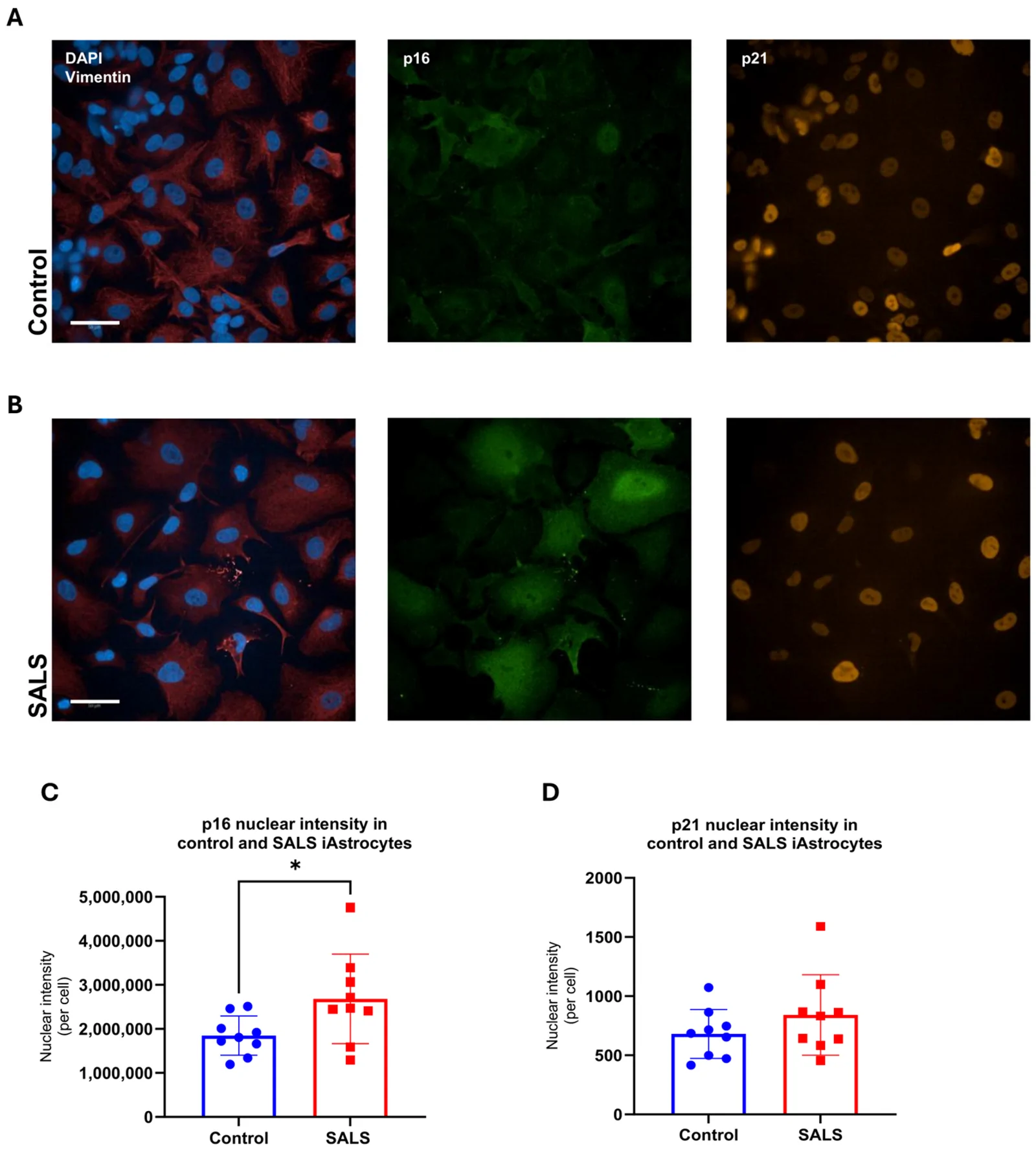
**p16 and p21 nuclear intensity in control and SALS-iAstrocytes.** Representative images of DAPI (blue), Vimentin (red), p16 and p21 staining in (**A**) control and (**B**) SALS-iAstrocytes. Scale bars represent 50 µm. (**C**) p16 nuclear intensity in control and SALS-iAstrocytes. (**D**) p21 nuclear intensity in control and SALS-iAstrocytes. Data presented as mean and standard deviation of three biological replicates from three control and three SALS-iAstrocytes. Analysis performed using Welch *t*-test (**C**) or unpaired *t*-test (**D**). * *p* ≤ 0.05. Where *p* value is not indicated, results were non-significant.

**Figure 8:**
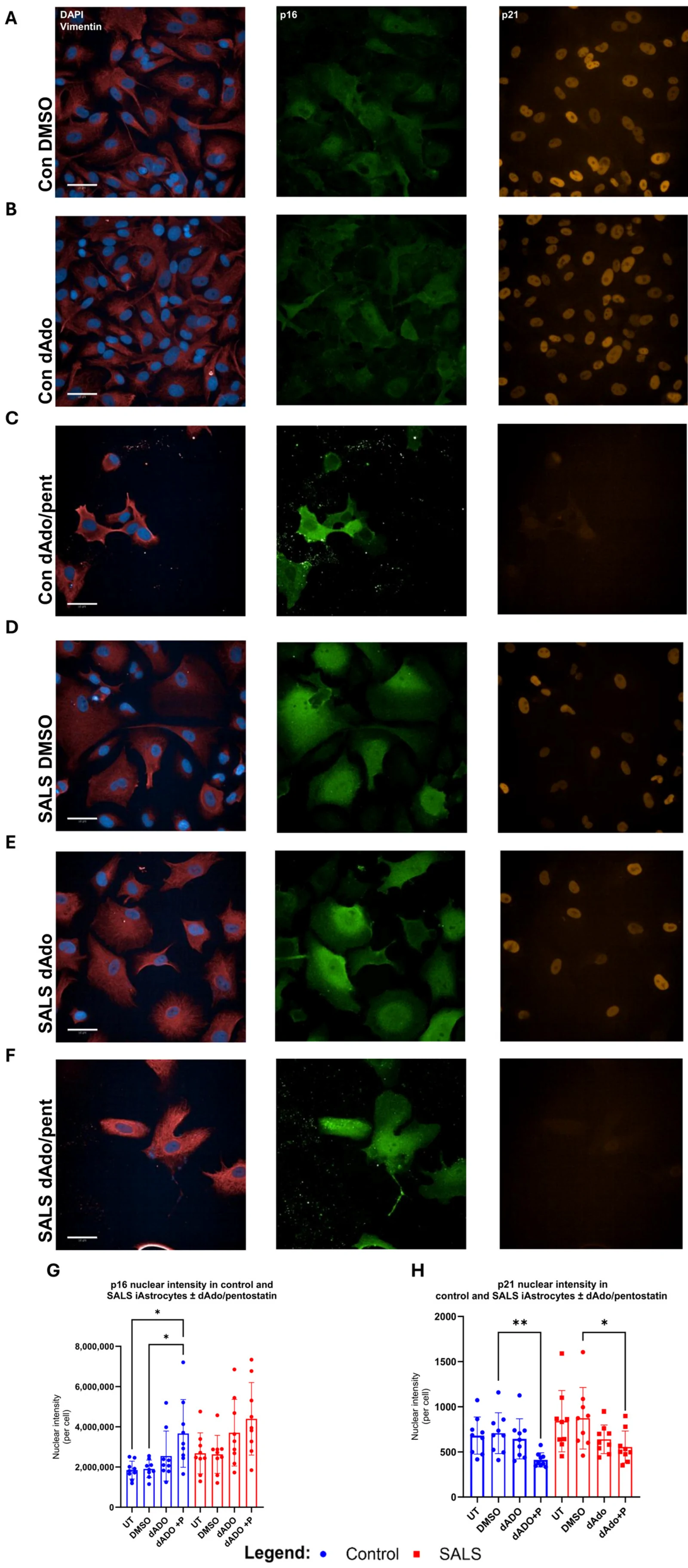
**The effect of deoxyadenosine stimulation on p16 and p21 nuclear intensity when ADA is inhibited.** Representative images of DAPI (blue), Vimentin (red), p16 and p21 staining in (**A**–**C**) control and (**D**–**F**) SALS-iAstrocytes cultured in (**A**,**D**) 0.05% DMSO, (**B**,**E**) 2 mM deoxyadenosine (dAdo) or (**C**,**F**) 2 mM dAdo and 0.05µM pentostatin. Scale bars represent 50 µm. (**G**) p16 nuclear intensity in control and SALS-iAstrocytes. (**H**) p21 nuclear intensity in control and SALS-iAstrocytes. Data presented as mean and standard deviation of three biological replicates from three control and three SALS-iAstrocytes. Analysis performed using a Kruskal–Wallis test or ordinary one-way ANOVA with a Bonferroni post-test within the control or SALS groups. * *p* ≤ 0.05, ** *p* ≤ 0.01. Where *p* value is not indicated, results were non-significant.

With these data in mind, we assessed the levels of two key markers of cellular senescence: nuclear levels of the early-stage p53 target p21 and the late-stage senescent marker p16. Immunofluorescence analysis showed that p21 levels were unchanged in SALS-iAstrocytes, whilst p16 levels were significantly elevated (Figure 7), suggesting a late-stage senescent phenotype. To assess whether this increase in p16 was linked to ADA function, we stimulated the purine pathway with deoxyadenosine in the absence or presence of the ADA non-reversible inhibitor pentostatin, an approach taken previously by our laboratory with adenosine that leads to increased astrocyte-mediated toxicity towards motor neurons [10]. Deoxyadenosine stimulation in controls brought p16 levels up to the level of SALS-iAstrocytes without stimulation, suggesting that the raised p16 levels in SALS-iAstrocytes under basal levels were purine metabolism-mediated via lack of ADA (Figure 8E). This was confirmed when stimulating the cells with adenosine when ADA was inhibited, which led to a significant increase in p16 levels in controls and a further increase in SALS-iAstrocytes (Figure 8E). Interestingly, deoxyadenosine stimulation in the presence of pentostatin led to a significant reduction in p21 nuclear levels in both control and SALS-iAstrocytes, perhaps indicative of a switch to a late-stage senescent phenotype.

DNA damage and senescence are closely tied, with the DNA damage response precipitating senescence; levels of γH2AX phosphorylation and 53BP1 regulate progression of the cell cycle and promote arrest if DNA damage levels are high [40,41,42]. Moreover, loss of ADA, as we have previously shown, leads to a build-up of dATP levels [14], which, if unchecked, can lead to inhibition of the ribonucleotide reductase (RNR) enzyme, impacting DNA repair, and can be propagated by adenosine [41]. γH2AX and 53BP1 levels were used as markers of DNA damage and repair. γH2AX is a marker of DNA double-strand breaks, and 53BP1 is used as a marker of the DNA damage response, localising to sites of double-stranded DNA breaks in astrocytes [42]. Only cells with nuclei containing five or more puncta of 53BP1 or γH2AX were counted, providing a rigorous cut-off that ignored background DNA damage events unrelated to treatment (Supplementary Figure S10).

**Figure 9:**
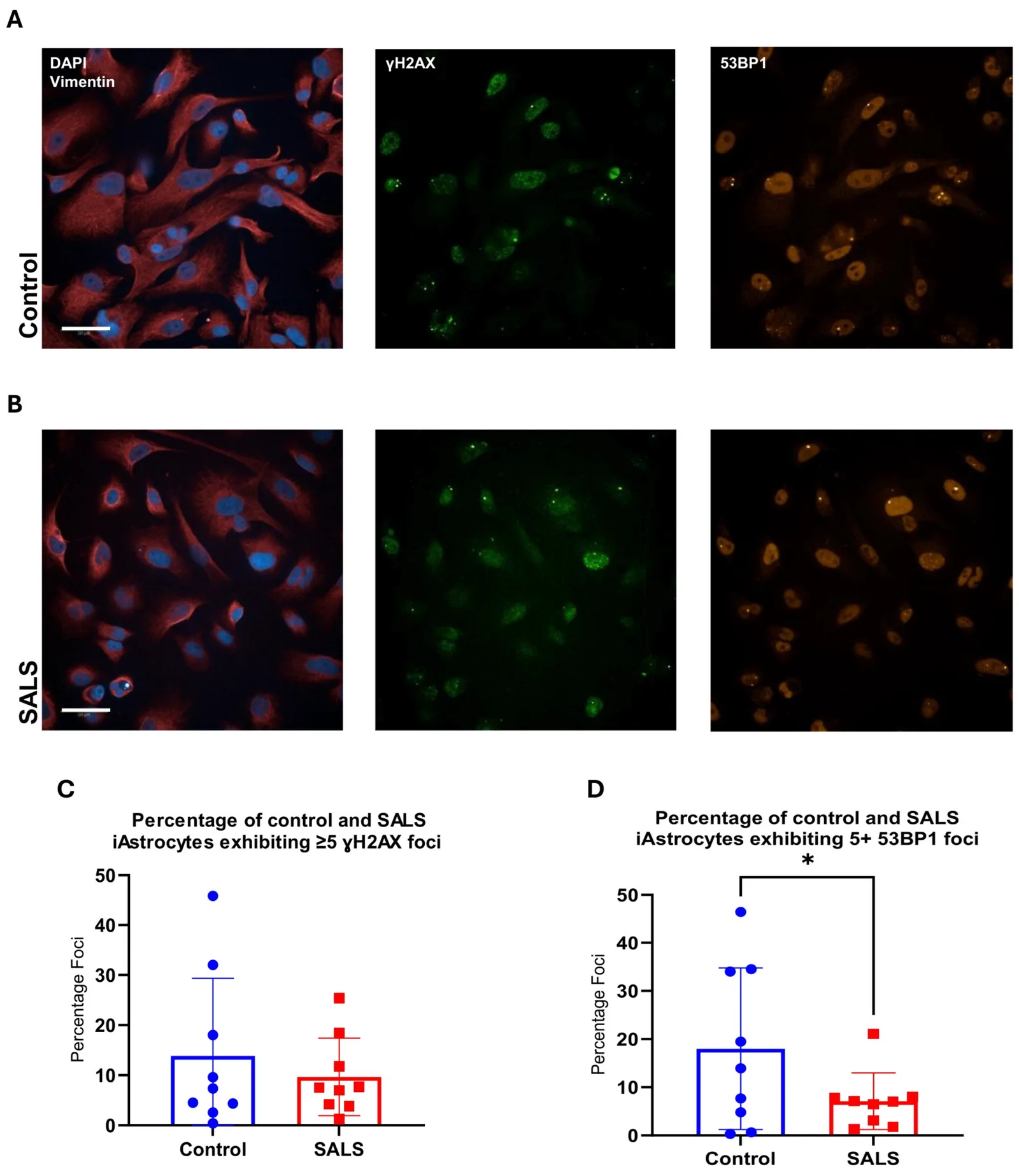
**γH2AX and 53BP1 foci levels in control and SALS-iAstrocytes.** Representative images of DAPI, Vimentin, γH2AX and 53BP1 staining in (**A**) control and (**B**) SALS-iAstrocytes. Scale bars represent 50 µm. (**C**) Percentage of iAstrocytes expressing ≥5 γH2AX foci in control and SALS-iAstrocytes. (**D**) Percentage of iAstrocytes expressing ≥5 53BP1 foci in control and SALS-iAstrocytes. Data presented as mean and standard deviation of three biological replicates from three control and three SALS-iAstrocytes. Data were first transformed with X = 1/Y followed by x = logit(Y) and analysed using an unpaired *t*-test (C and D). * *p* ≤ 0.05. Where *p* value is not indicated, results were non-significant.

**Figure 10:**
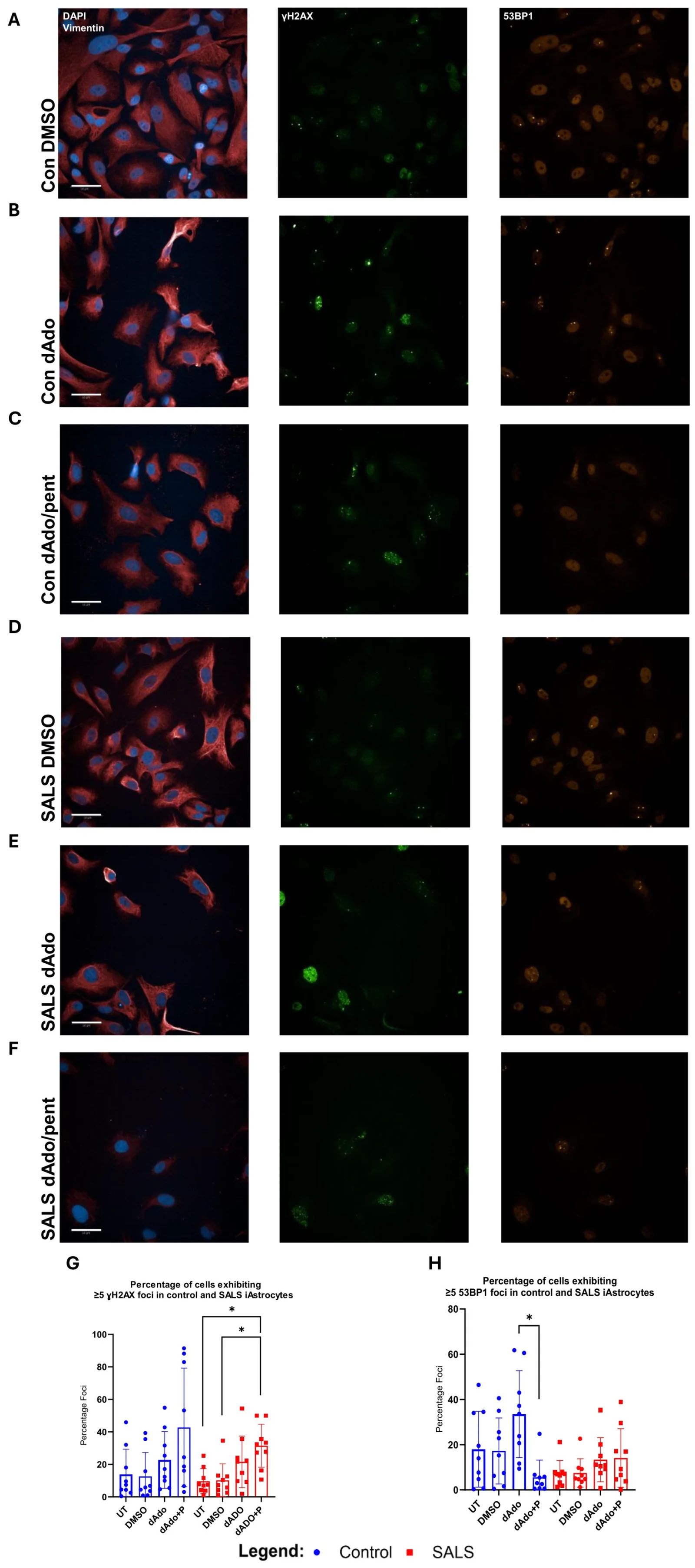
**The effect of deoxyadenosine stimulation on γH2AX and 53BP1 foci levels in iAstrocytes when ADA is inhibited.** Representative images of DAPI, Vimentin, γH2AX and 53BP1 staining in (**A**–**C**) control and (**D**–**F**) SALS-iAstrocytes cultured in (**A**,**D**) 0.05% DMSO, (**B**,**E**) 2 mM deoxyadenosine (dAdo) or (**C**,**F**) 2 mM dAdo and 0.05µM pentostatin. Scale bars represent 50 µm. (**G**) Percentage of iAstrocytes expressing ≥5 γH2AX foci in control and SALS-iAstrocytes. (**H**) Percentage of iAstrocytes expressing ≥5 53BP1 foci in control and SALS-iAstrocytes. Data presented as mean and standard deviation of three biological replicates from three control and three SALS-iAstrocytes. Data were first transformed with X = 1/Y, followed by x = logit(Y) and analysed using an ordinary one-way ANOVA within the control or SALS groups * *p* ≤ 0.05. Where *p* value is not indicated, results were non-significant.

Under basal conditions, 53BP1 levels were lower in SALS-iAstrocytes, whereas γH2AX levels were unchanged (Figure 9). When control iAstrocytes were stimulated with deoxyadenosine, a trend for an increase in 53BP1 was observed that was significantly reduced to SALS untreated levels when ADA was inhibited (Figure 10H). This loss of DNA repair led to an increase in γH2AX levels, which failed to reach significance (*p* = 0.11) but suggested that ADA inhibition in astrocytes leads to increased DNA damage in the presence of high levels of deoxyadenosine due to loss of 53BP1 (Figure 10G,H). Deoxyadenosine treatment with further ADA inhibition in SALS-iAstrocytes had no effect on 53BP1 levels but led to a significant increase in γH2AX levels, suggesting that SALS-iAstrocytes are especially susceptible to elevated DNA damage due to loss of ADA.

## 3. Discussion

In this study, we have continued our previous work, which identified loss of ADA in SALS-iAstrocytes and showed that ADA loss was linked to purine metabolism dysfunction, which was not only observed in vitro, but ex vivo and in SALS patient-derived biofluids. Our data suggest that TDP43 dysfunction drives ADA-mediated purine metabolism dysfunction in vitro, with downstream consequences including DNA damage due to DNA repair inhibition and concomitant cellular senescence. Furthermore, our data suggest that targeting the ADA pathway may be beneficial in slowing down disease progression.

Our initial Western analysis demonstrated that, as with C9orf72-iAstrocytes, SALS-iAstrocytes show loss of the enzyme CD73 (Figure 1), which dephosphorylates adenosine nucleotides to produce adenosine. The cause of this common alteration and whether it is a pathogenic or protective mechanism, preventing toxic adenosine accumulation due to loss of ADA, is unclear. If protective, carbon could be shuttled via AMP to ADP (both intracellularly and extracellularly), which was elevated in untreated C9orf72-iAstrocytes but not in SALS-iAstrocytes (Figure 2J). However, stimulation of the pathway with inosine led to a significant increase in ADP levels in SALS-iAstrocytes (Figure 2R), suggesting a preference for carbon flow via AMP. Interestingly, in control-iAstrocytes, inosine supplementation led to an increase in the ATP:ADP ratio, which was not observed in SALS-iAstrocytes, indicating further disruption to energy production (Figure 2S). Unlike in C9orf72-iAstrocytes, HGPRT levels were reduced compared to controls in SALS-iAstrocytes (Figure 1). HGPRT converts hypoxanthine to IMP or guanosine monophosphate (GMP) and is the key enzyme in purine salvage and thus the principal source of purine nucleotides. This suggests reduced purine salvage was occurring in these cells, although a loss of IMP was not observed (Supplementary Figure S2E). However, IMP production occurs via multiple pathways including de novo synthesis (PDNS) [43,44,45,46].

In our evaluation of the PDNS enzymes, although we were able to see some potential trends for a reduction in the levels of some of the targets, including ATIC, no widespread dysfunction was observed, including with PAICS, which has been recently implicated in C9orf72 and SALS [22]. The reasons for this may be model-specific, as we did not see any changes in PAICS levels in C9orf72-iAstrocytes in our previous study. Under normal physiological conditions, purines are mainly supplied through the salvage pathway. The de novo pathway is typically activated only when the demand for purines surpasses the capacity of the salvage system to meet this need [47]. Moreover, purine salvage and PDNS levels are known to differ between astrocytes and neurons both from an expression level point of view and spatially [48,49]. With this in mind, our PAICS-targeted purinosome work in C9orf72-iAstrocytes showed a loss of purinosome levels, likely caused by DPR interaction with PAICS, FGAMS, ADSL and ATIC, which led to mitochondrial functional defects when mTOR was inhibited [14]. Although the Singh et al. study did not measure purinosome or metabolic function, they suggested that loss of PAICS may be mTOR-mediated. From a technical viewpoint, we were unable to measure purinosome formation in SALS-iAstrocytes to assess whether the C9orf72 and SALS-iAstrocytes show the same dysregulation. However, it is clear that our data align with the Singh et al. study and that targeting PDNS/purine metabolism represents a novel therapeutic approach in ALS. With this in mind, inosine has been shown to activate mTOR in cells undergoing nutrient stress via the transcription factor specificity protein 1 [50]; alterations of adenosine receptor 2A function can influence purinosome formation [51,52], which is also susceptible to age-associated metabolic alterations [53,54].

Functionally, as already described, SALS-iAstrocytes sit between control and C9orf72-iAstrocytes in terms of purine metabolite levels, ADA enzyme activity and levels of dNTPs (Figure 2), which is clearly linked to the level of ADA. These effects were not only observed in vitro but also in the CSF of SALS and C9orf72 cases (Figure 3). This provides strong evidence that the in vitro findings observed are not cell model effects and represent a snapshot of the SALS cohort as a whole, which, as we have shown, has variable levels of purine metabolites downstream of ADA, which are linked to age and sex and potentially linked to ALS disease progression (Figure 3 and Figure 4). Age-related changes in purine levels have been reported previously in similar metabolomic approaches using human subjects, especially in the context of hypoxanthine, xanthine and urate [55,56].

From an ALS point of view, what is interesting from our data set is that this ageing and sex effect is much more evident when focusing on age at death rather than when taking age at onset into account. This is evident by the clear positive correlation between downstream ADA metabolites inosine, hypoxanthine and xanthine and length of disease progression in female ALS cases (Figure 4). This suggests that targeting the purine metabolism pathway post-symptom onset could extend the life of people with ALS. However, mechanistically, this is not due to the production of urate, which has been linked to ALS survival previously [57,58]. CSF urate levels did not correlate with disease progression, suggesting that the beneficial effect of elevated purine levels is not endpoint urate production but driving carbon to the purine salvage pathways and/or metabolic pathways, providing a beneficial effect that is influenced by ageing and sex. This aligns with our previous study, in which we performed inosine supplementation assays [10]. All C9orf72/SALS-iAstrocytes produced more uric acid when supplemented with inosine, but only those ALS-iAstrocytes that showed increased metabolic function with inosine showed increased support towards motor neurons in co-culture. Moreover, although the production of urate may confer neuroprotective effects, including as an antioxidant [59,60], with some studies pointing towards a positive relationship between serum uric acid concentrations and ALS disease progression/survival rates in males [57,58], previous inosine clinical studies in ALS cases using urate as a target engagement measure did not show efficacy [61]. Future work in this area should take into account different target engagement measures such as CSF hypoxanthine/xanthine levels and use of age and sex as a way to stratify ALS cases. Evidence suggests that ADA levels can be influenced by 17β-oestradiol (E2), an oestrogen hormone known to regulate enzymes involved in purine and pyrimidine biosynthesis pathways. In MCF-7 human breast cancer cells, E2 treatment stimulates ADA mRNA expression, an effect that can also be replicated using tamoxifen, a drug commonly used in breast cancer therapy [62]. Recent evidence indicates that oestradiol may play a protective role in ALS, particularly in pre-menopausal women [63]. Additionally, greater lifetime exposure to endogenous oestrogen has been associated with extended survival in ALS patients [64], while treatment with 17β-oestradiol has demonstrated protective effects in SOD1 mouse models [65]. Oestradiol has also been shown to mitigate demyelination and axonal damage in mouse models of multiple sclerosis (MS) [66] and may confer neuroprotection in other neurodegenerative conditions [67]. As myelinated axons are predominantly located in the white matter and white matter alterations have been observed in ADA deficiency [68], this may go some way to explain our motor cortex white vs. grey matter differences and why ADA levels correlate with age at death. Future research could therefore explore the role of oestradiol in regulating ADA and how this interaction may contribute to its effects in ALS, especially in oligodendrocytes, which are highly prevalent in white matter.

A focus on CSF and not blood or plasma is evident from the plasma data presented in this study, which show the opposite of what is observed in the CSF. At the group level, no changes were observed between controls and SALS/C9orf72 cases. However, our data suggested that plasma purine levels downstream of inosine negatively correlated with age and, to a greater extent, in SALS cases compared to controls (Figure 5 and Figure S6). Again, this effect was more evident post-symptom-onset in SALS cases but, unlike in the CSF, was driven by male rather than female cases. However, no link between plasma purine levels and disease progression was observed in males or females.

As well as the purine salvage and the metabolic benefits of increased ADA activity, ADA activity is important for 53BP1-mediated DNA repair mechanisms. Our data suggest a senescence phenotype in the SALS cases, evident from increased p53β, increased p16 levels, and decreased 53BP1 levels, which could be mimicked in controls by inhibiting ADA with pentostatin and stimulating with deoxyadenosine (Figure 7, Figure 8, Figure 9 and Figure 10). Pentostatin treatment has been previously shown to affect memory, mood and neurotoxicity in a dose-dependent manner [69]. Although Schneider et al. [70] reported that astrocytes are capable of expressing γH2AX without a detectable increase in 53BP1, the control astrocytes used in this study were able to efficiently upregulate 53BP1 nuclear puncta in the presence of deoxyadenosine. However, this ability was attenuated once ADA activity was inhibited. Since SALS-iAstrocytes have an inherent loss of ADA activity, their inability to produce a similar response to control lines with or without ADA inhibition further confirms our theory that ADA activity is essential to upregulate the DNA damage response. The mechanism for this, as previously suggested, is a build-up of dNTP levels and/or dysfunction in the methionine cycle/folate pathway caused by deoxyadenosine in the absence of ADA. Work in the breast cancer field has shown that inhibiting ADA with dipyridamole in the presence of the synthetic antifolate 3-O-(3,4,5-trimethoxybenzoyl)-(−)-catechin inhibited the recruitment of 53BP1 to DNA double-strand breaks by disrupting both the folate cycle and the methionine cycle, sensitising cancer cells to radiotherapy [71]. Disruption to the methionine cycle would also affect DNA methylation, which is a known pathological mechanism in ALS that we previously postulated would be negatively affected by loss of ADA activity [20].

In addition to regulating deoxyadenosine levels, ADA is crucial for removing excess adenosine from cells, which, as we have shown previously, causes motor neuron death when ADA is inhibited [10]. As we previously discussed in that publication, adenosine levels can rise both intracellularly and extracellularly due to bidirectional nucleoside transporters and the release of ATP, especially under neuronal stress. This can lead to adenosine receptor activation and, in astrocytes, loss of glutamate clearance. This would suggest that adenosine receptor antagonism (in addition to maintaining ADA levels) would be beneficial in ALS. Recent data published since our original 2019 paper suggest that this is the case, with an ongoing clinical trial due to be published at the time of writing [72,73]. Interestingly, a recent study was published suggesting that the adenosine receptor antagonist caffeine can influence cognitive performance in ALS cases with an rs2472297 single nucleotide polymorphism in Cytochrome P450 Family 1 Subfamily A Member 1 [74]. We found that caffeine levels were lower in C9orf72-iAstrocytes compared to controls, with SALS-iAstrocytes again lying between controls and C9orf72 cases (Supplementary Figure S2F, [14]), which could exacerbate the adenosine effect described above. The same effect was not observed at the group level in SALS CSF, and no significant correlation with disease progression was observed (Supplementary Figure S12). In the CSF and plasma, no differences in adenosine levels were observed at the group level between controls and SALS cases. Moreover, no significant correlation was observed between age or disease progression and CSF adenosine levels in SALS, or age in controls (Supplementary Figures S11 and S12). A significant positive correlation between plasma adenosine levels and SALS age at onset was observed, which was driven by females (Supplementary Figure S11G,H). However, this significance in plasma was lost when taking into account age at death, and no disease progression correlation was observed. The reason for this onset correlation is unclear due to the multifactorial nature of adenosine metabolism. However, this correlation was not observed in control cases, suggesting a disease-specific effect.

With the reduction of CD73 in SALS-iAstrocytes in mind, work in the Parkinson’s disease field has shown that increasing ADA levels, adenosine receptor antagonism or CD73 inhibition protects against 6-Hydroxydopamine in vitro and in vivo [75]. Furthermore, in a β-amyloid model of Alzheimer’s disease, CD73 inhibition decreased synaptic potentiation, reflected by impaired long-term potentiation in mouse hippocampal slices, linking CD73 to adenosine receptor 2A function [76]. These data suggest that the decrease in CD73 observed in SALS-iAstrocytes may indeed be protective. However, measurement of the intracellular 5′-nucleotidase is required to start to uncover the full mechanistic picture, including why CD73 is reduced in both C9orf72- and SALS-iAstrocytes, whilst HGPRT is increased in C9orf72 but reduced in SALS-iAstrocytes compared to controls.

Finally, as well as focussing on downstream consequences of ADA dysfunction, we looked at the upstream causes. In our previous publication, we showed that DPRs driven by C9orf72 hexanucleotide expansion cause the loss of ADA, leading to purine dysfunction in C9orf72-ALS. However, this would not be the case in sporadic ALS, with a common factor between C9orf72-ALS and sporadic ALS being TDP43 pathology. Cytoplasmic TDP aggregation can be formed by the 35 kDa TDP43 fragment recruiting the full-length TDP43 to the cytoplasm, removing it from the nucleus [77]. The levels of this 35 kDa fragment were evident in the iAstrocyte model used in this study and elevated in SALS and C9orf72-iAstrocytes compared to controls (Figure 6). SOD1-iAstrocytes did not show the same level of TDP43 pathology and, furthermore, did not show loss of ADA, suggesting a link between TDP43 pathology and ADA levels, which was somewhat confirmed by correlation analysis in the iAstrocyte cohort, which suggested that the greater the TDP43:35 ratio, the higher the level of ADA (Figure 6D). Arsenite treatment of SY5Y cells, which is a widely used approach to promote aggregation and mislocalisation of TDP43, also led to a significant loss of ADA at the protein level and the mRNA level (Figure 6), again suggesting a possible link between TDP43 pathology and loss of ADA. Loss of ADA both at the protein and RNA levels aligned with our previous iAstrocyte data, which show loss of ADA at the protein and RNA levels in both C9orf72- and SALS-iAstrocytes [10]. However, this effect was not observed in two TDP43 fly models of ALS. The reasons for this are unclear and may be model- or cell-type-specific; as in our previous publication, DPR1000 flies expressing poly-GR and poly-PR had lower levels of ADA [14].

In this article, we have built upon our previous work on purine metabolism in SALS-iAstrocytes and have linked ADA levels with purine metabolism dysfunction, DNA damage, senescence and TDP43 pathology. Our data support the hypothesis that ADA levels lead to purine metabolism alterations, with lower levels typically showing greater dysfunction. Moreover, our data highlight that ADA activity is important for mitigating DNA damage through the action of 53BP1, which is linked to senescence. These purine alterations are not just observed in vitro but also in biofluids from SALS cases and suggest that targeting the CNS ADA-mediated purine metabolism/salvage pathway may be protective in ALS and is influenced by age and sex. Further work is required to elucidate the exact TDP43-related mechanisms that lead to loss of ADA and how these affect purine metabolism, purine salvage and the PDNS.

## 4. Materials and Methods

### 4.1. Ethics

All ethical approvals were in place, and subject informed consent was obtained for all patient-derived cell cultures (study numbers STH16573, STH16350, Research Ethics Committee (REC) reference: 12/YH/0330). All ethical approvals were in place for the use of the patient-derived plasma/CSF data under the study title: A Multicentre Biomarker Study in Neurodegeneration (REC reference: 16/LO/2136, IRAS project ID: 204405). Samples were collected at the Sheffield Teaching Hospital, and all investigators were blinded to personal donor information outside clinical disease data. Postmortem tissues came from Sheffield Brain Tissue Bank (REC reference: 08/MRE00/103+5, IRAS project ID: 140226). Further information can be found in the Institutional Review Board Section. *Drosophila melanogaster* is not legislated and is outside the NC3Rs restriction; however, all applicable international, national, and/or institutional guidelines for the care and use of animals were followed.

### 4.2. Human Biosamples

iNPC reprogramming was performed on skin biopsies from three controls, three SALS cases, three C9orf72-ALS cases and two SOD1 cases with an A4V mutation, using the method developed by Meyer et al. [12,78]. The control average age at biopsy was 58 (±15.7) years. SALS average age at biopsy was 45.6 (±16) years, and average disease duration was 61 (±35.8) months (Supplementary Table S1). C9orf72-ALS case average age at biopsy was 60.7 (±9.2) years, and average disease duration was 26 (±6.2) months. SOD1 average age at biopsy was 51.5 (±16.2) years, and average disease duration was 150 (±70.7) months (Supplementary Table S1). CSF was taken from a total of 38 controls and 81 SALS cases. Average age at time of donation for controls was 55.3 (±14.4) years, average age at onset for SALS cases was 59.3 (±14.5) years, average age at death was 63.7 (±13.5) years, and disease duration was 36.1 (±21.2) months. Plasma was taken from 38 controls and 79 SALS cases. Average age at time of donation for controls was 57.4 (±14.5) years, average age at onset for SALS cases was 60.7 (±14.2) years, average age at death was 64 (±13.4) years, and disease duration was 39.2 (±21.2) months. All data were compared to CSF from 7 C9orf72-ALS cases and plasma from 10 C9orf72-ALS cases, as previously described [14]. Post-mortem white and grey matter were taken from a total of 12 controls with an average age at autopsy of 62.8 (±9.2) years (Supplementary Table S2). SALS white matter was taken from a total of 10 patients with an average age at autopsy of 53.8 (±8.9) years and average disease duration of 34.4 (±23.2) months (Supplementary Table S3). SALS grey matter was taken from 9 patients with an average age at autopsy of 54.0 (±9.5) years and average disease duration of 35 (±22) months. Existing C9orf72 post-mortem data were used as a comparison, as originally published in [14].

### 4.3. Tissue Culture

iNPC lines were generated as described previously and have been extensively characterised by our group and others [10,11,12,14]. Briefly, iNPCs were cultured in DMEM containing 1% N-2 supplement (Gibco, Paisley, Scotland, Thermo Fisher Scientific, Loughborough, UK), 1% B27 (Gibco), and 20 ng/mL fibroblast growth factor-2 (Preprotech, Cranbury, NJ, USA). Differentiation into iAstrocytes was induced via the introduction of DMEM containing 25 mM glucose, 10% heat-inactivated FBS (Sigma-Aldrich, St. Louis, MO, USA) and 0.2% N-2 supplement. Prior to collection for metabolomics, cultures were supplemented with 7.5 mM inosine for 24 h. For senescence and DNA damage immunofluorescence assays, iAstrocytes were cultured in either 0.05% DMSO, 2 mM dAdo and/or 0.5 µM pentostatin for 24 h prior to fixing. Human SH-SY5Y neuroblastoma cells (ATCC, sourced via Sigma-Aldrich) were cultured in Dulbecco’s Modified Eagle Medium/Nutrient Mixture F-12 (DMEM/F-12) supplemented with 10% (*v*/*v*) foetal bovine serum (FBS) and 1% penicillin-streptomycin (all reagents were from Thermo Fisher Scientific). Cells were maintained in a humidified incubator at 37 °C with 5% CO_2_. For experimental treatments, cells were seeded into 24-well plates at a density of 100,000 cells per well. Following a 24-h incubation period to allow for cell adherence, oxidative stress was induced by treating the cells with 500 µM sodium arsenite (NaAsO_2_) for 1 h. After the treatment, the arsenite-containing medium was aspirated, and the cells were washed once with fresh, pre-warmed culture medium to ensure the complete removal of residual stress. Cells were then incubated in fresh medium for up to a 6 h recovery period prior to downstream analysis. All cells were maintained at 37 °C, 5% CO_2_ and 95% humidity. Cell viability and proliferation were assessed using the Incucyte^®^ S3 Live-Cell Analysis System (Sartorius, Göttingen, Germany). Cell density was quantified from bright-field images using Phase Object Count analysis [79].

### 4.4. Western Blotting

Western blotting was carried out as described previously for iAstrocytes [14]. Briefly, cells were lysed using RIPA, urea, or IP buffer containing protease and phosphatase inhibitors prior to protein quantification via Bradford assay. Laemmli buffer was then added to the sample before 10–30 µg protein was loaded into polyacrylamide gels and resolved by SDS-PAGE. Proteins were transferred to either nitrocellulose or polyvinylidene membranes and incubated overnight at 4 °C in primary antibody. Membranes were washed in TBST, and a secondary antibody was added for 1 h at RT before imaging with EZ-ECL HRP chemiluminescence kit on an Odyssey Fc imaging system (LI-COR, Lincoln, NE, USA). Densitometry was carried out with Image Studio Lite 5.2 (LI-COR). Details of the antibodies used can be found in Tables S4 and S5.

### 4.5. RT-qPCR

RT-qPCR was carried out as described previously [14]. Briefly, RNA was extracted with TRIzol reagent, followed by chloroform phase separation and isopropanol precipitation. RNA was DNase-treated, and RNA concentration was quantified on a NanoDrop 1000 spectrophotometer (Thermo Fisher Scientific). cDNA was synthesised from 2 µg RNA using M-MLV reverse transcriptase (Thermo Fisher Scientific). For the SH-SY5Y cells, total RNA was isolated from the cells using either the GenElute Mammalian Total RNA Miniprep Kit (Sigma-Aldrich) or Qiazol Lysis Reagent (Qiagen, Manchester, UK), strictly adhering to the respective manufacturer’s instructions. RT-qPCR was performed with Ultra-Fast SYBR Green (Agilent, Santa Clara, CA, USA) on a CFX96 RealTime System C1000 Touch Thermal Cycler (Bio-Rad Laboratories, Hercules, CA, USA), with analysis carried out using CFXMaestro v2.2 (Bio-Rad). Primer sequences provided in Table S6.

### 4.6. Metabolomics

#### 4.6.1. iAstrocytes

iAstrocyte LC-MS was carried out as described previously [14]. Briefly, iAstrocytes were harvested in ice-cold methanol before dehydration under vacuum. Dehydrated pellets were resuspended in methanol and water (1:1 *v*/*v*), agitated and centrifuged prior to analysis using a Synapt G2Si high-resolution TOF-MS coupled to an Acuity UPLC system (Waters, Wilmslow, UK) equipped with a BEH C18 column (2.1 × 50 mm, 1.7 µm particle size; Waters). The mobile phases consisted of water with 0.1% formic acid and acetonitrile with 0.1% formic acid at a flow rate of 0.3 mL/min. Samples were analysed using electrospray ionisation in both negative and positive modes. Data processing was performed with XCMS Online [80], as outlined in Parker et al. [81]. Data were normalised to pellet weight and analysed using Metaboanalyst v6.0 [81], with pathway analysis performed using the mummichog algorithm [82,83]. All data are freely available on the University of Sheffield data repository at https://doi.org/10.15131/shef.data.30739946.

#### 4.6.2. CSF and Plasma

CSF and plasma were part of the AMBROSIA (A Multicentre Biomarker Resource Strategy In ALS) cohort and harvested as described by Thompson et al. [84]. UPLC-MS/MS was carried out by Metabolon, Inc. as described previously [14]. Samples were prepared using the automated MicroLab STAR system (Hamilton Company, Reno, NV, USA). Recovery standards were added to samples prior to extraction, and proteins were precipitated with methanol under agitation and centrifuged before evaporation and overnight storage under nitrogen. Metabolomics was carried out using a Q-Exactive Orbitrap mass spectrometer (Thermo Fisher Scientific) coupled to an Acquity UPLC system (Waters) with heated electrospray ionisation operated at 35,000 resolution. Analysis was performed using four complementary methods [85], two reverse-phase methods with positive electrospray ionisation, one reverse-phase method with negative ionisation and one hydrophilic interaction chromatography method with negative ionisation. Samples for QC were pooled matrix samples, extracted water blanks and internal QC standards run in conjunction with experimental samples injected at randomised intervals. Median relative standard deviation calculations were performed for both instrument and overall process variability as QC metrics. Data processing was performed using Metabolon, Inc-developed applications, with compound identification through comparison against proprietary libraries of authentic chemical standards. Run-day normalisation was implemented by adjusting compound medians to 1.0. All data were tested for normality distribution prior to correlation with age at death in years, age at symptom onset in years or disease progression in months, which was calculated as time from symptom onset to death. Control age was that at the time of biopsy. All data were analysed by Pearson’s or Spearman’s correlation analysis.

### 4.7. ADA Activity

ADA activity and inosine output were assessed using a colorimetric ADA activity assay kit (Abcam, Cambridge, UK) as described previously [14]. Briefly, iAstrocyte pellets were lysed in an appropriate volume of ADA activity assay buffer containing 10% protease inhibitor cocktail and protein concentration was determined using a Bradford assay. An inosine standard was generated (1 in 2 serial dilution, 50.0–0.78 nmol/well) and loaded into the supplied 96-well UV-transparent plate. Samples were then loaded into wells at a protein concentration of 10 µg/well. A reaction mix comprising ADA assay buffer, converter mix E, xanthine enzyme mix and ADA substrate was added to half the sample-containing wells, whilst a background control mix comprising ADA assay buffer, converter mix E and xanthine enzyme mix was added to the standard wells and the remaining sample wells. The plate was then incubated at 37 °C for 5 min prior to measuring absorbance (OD = 293 nm) at 37 °C in 30 s intervals for 60 cycles using a PHERAstar plate reader (BMG Labtech, Ortenberg, Germany). Inosine output was calculated by taking the OD reading at a given time point and removing background OD before calculating nmol inosine/well (as determined by the standard curve) and normalising to protein concentration. ADA activity was calculated by taking the inosine output from two timepoints (T1 and T2) and entering that into the following formula:

ADA activity = (ΔI/ΔT × M) × D

where:

ΔI = Inosine output at T2 − Inosine output at T1

ΔT = T2 − T1

M = protein concentration/well

D = dilution factor in well

### 4.8. dNTP Measurement by Coupled Click Chemistry and DNA Polymerase-Based Assay

The dNTP measurement in iAstrocytes was carried out as described previously [14]. iAstrocytes were harvested in methanol before dehydration under vacuum. Purine dNTP quantification was performed as described by Huang et al. [86]. First, biotinylated oligonucleotide primers specific to dGTP and dATP were incubated with cell extracts in the presence of DNA polymerase and EdUTP to facilitate nucleotide-dependent incorporation. Biotin-labelled DNA products were extracted by incubating samples with Streptavidin beads under agitation. Extracted DNA was conjugated with the TAMRA fluorophore through copper(I)-catalysed alkyne-azide cycloaddition. Fluorescence was measured with 529/575 nm Ex/Em wavelengths using the Tecan Spark 10M (Tecan Group Ltd., Männedorf, Switzerland).

### 4.9. Immunofluorescence

Immunofluorescence assays were carried out as described previously [14]. Briefly, iAstrocytes were plated in black-walled 96-well plates (Greiner, Kremsünster, Austria) prior to fixing with paraformaldehyde and permeabilisation with Triton X-100. Cells were then blocked and incubated with primary antibody overnight at 4 °C. Cells were washed and incubated with secondary antibody for 1 h at RT before the addition of Hoechst. Imaging was carried out using the Opera or Operetta High-Content Imaging systems (PerkinElmer, Waltham, MA, USA) and analysis with Harmony High-Content Imaging and Analysis software (PerkinElmer, v5.2). Details of antibodies used can be found in the Supplementary Materials and Methods (Supplementary Tables S4 and S5).

### 4.10. TDP43 Drosophila melanogaster

*Drosophila* were raised on cornmeal–yeast–soya flour medium (80 gm/L medium cornmeal, 18 gm/L dried yeast, 10 gm/L soya flour, 80 gm/L malt extract, 40 gm/L molasses, 8 gm/L agar, 0.25% nipagin, 0.4% propionic acid) at 25 °C under a 12 h light:dark cycle. Canton-S (RRID: BDSC_64349), w^1118^ (RRID: BDSC_3605), TBPH^Δ23^ (RRID: BDSC_93599), TBPH^KO.hTARDBP.WT^ (RRID: BDSC_93125) and TBPH^KO.hTARDBP.M337V^ (RRID: BDSC_93126) were obtained from the Bloomington *Drosophila* Stock Center (BDSC). TBPH^Δ23^ carries a 1616 bp deletion that removes portions of the *TBPH* coding sequence and regulatory regions, resulting in a complete loss of endogenous TBPH protein expression [87]. The TBPH^KO.hTARDBP.WT^ and TBPH^KO.hTARDBP.M337V^ flies are endogenous knockouts replaced with wild-type or mutant human TDP43 [88]. The mutant flies have been shown to have increased levels of ubiquitinated proteins and phosphorylation of the TDP43 protein in neurons. In total, 10–15 flies (equal numbers of males and females), per repeat, were aged to 7 days post-eclosion, snap-frozen on dry ice, and RNA was extracted using TRIzol chloroform extraction as described previously [14].

### 4.11. Statistical Methodology

Statistical analyses were performed using GraphPad Prism software (v10.0.3). Data were first assessed for normality using the D’Agostino–Pearson omnibus test, Anderson–Darling test, Shapiro–Wilk test, and Kolmogorov–Smirnov test with the Dallal–Wilkinson–Lillie correction for *p*-values. Where appropriate, variance was evaluated using an F-test prior to conducting parametric or non-parametric analyses. The specific statistical tests applied are indicated in the figure legends. All error bars represent standard deviation. In this study, iAstrocyte lines were generated from three control individuals, three SALS patients, and two SOD1 patients, and compared to three C9orf72 patients. While it is common practice to average assay results from each cell line and treat these averages as biological replicates for statistical analysis, in this work, each assay was considered an independent biological replicate. This approach was adopted to more accurately reflect variability between replicates, including those derived from the same cell line, consistent with the methodology used in our previous studies and those of others.

## Data Availability

The data presented in this study are openly available in the University of Sheffield data repository at https://doi.org/10.15131/shef.data.30739946.

## Supplementary Materials

The following supporting information can be downloaded at: https://www.mdpi.com/article/10.3390/ijms27188236/s1.

## Abbreviations

The following abbreviations are used in this manuscript:

CAIR	5-aminoimidazole-4-carboxylate ribonucleotide
ATIC	5-Aminoimidazole-4-carboxamide ribonucleotide formyltransferase
PPAT	Amidophosphoribosyltransferase
ADA	Adenosine Deaminase
ADK	Adenosine Kinase
ADSL	Adenylosuccinate Lyase
ALS	Amyotrophic Lateral Sclerosis
C9orf72	Chromosome 9, Open Reading Frame 72
DNPB	De novo purine biosynthesis
DPR	Dipeptide Repeat Protein
CD73	Ecto-5′-nucleotidase
FGAMS	Formylglycinamide ribonucleotide amidotransferase
HRE	Hexanucleotide Repeat Expansion
HGPRT	Hypoxanthine-Guanine Phosphoribosyltransferase
iNPC	Induced Neural Progenitor Cell
iAstrocyte	Induced Neural Progenitor Cell-Derived Astrocyte
IMP	Inosine Monophosphate
PAICS	Phosphoribosylaminoimidazole carboxylase/phosphoribosylaminoimidazole succinocarboxamide synthetase
GART	Phosphoribosylglycinamide formyltransferase/phosphoribosylglycinamide synthetase/phosphoribosylaminoimidazole synthetase
PNP	Purine Nucleoside Phosphorylase
SAICAR	Succinylaminoimidazole carboxamide ribonucleotide
XO	Xanthine Oxidase
