# Supplementary material for: Adenosine Deaminase-Mediated Purine Dysfunction Leads to DNA Repair Inhibition and Senescence in Sporadic Amyotrophic Lateral Sclerosis
Source: Int J Mol Sci. 2026 Sep 16;27(18):8236. doi: 10.3390/ijms27188236 (PMC13607153; doi:10.3390/ijms27188236)
Supplement: Supplementary file 1 [file ijms-27-08236-s001.zip › Supplementary Figures-140926.pdf]

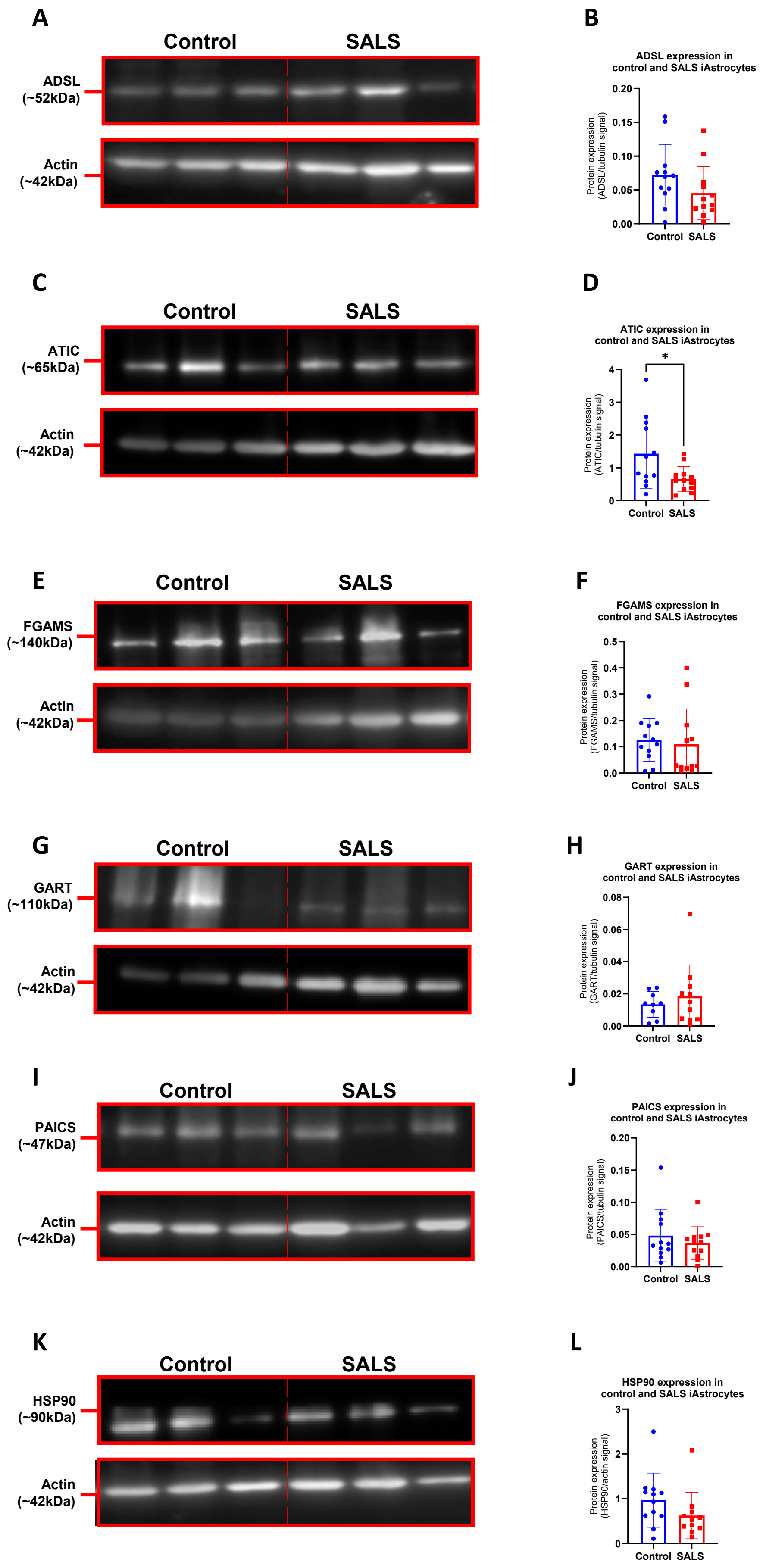

Supplementary Figure S1. DNPB enzyme expression. (**A**) Representative images and (**B**) densitometry analysis of adenylosuccinate lyase (ADSL) Western blot in control and SALS iAstrocytes. (C) Representative images and (**D**) densitometry analysis of 5-Aminoimidazole-4-carboxamide ribonucleotide formyl-transferase (ATIC) Western blot in control and SALS iAstrocytes. (**E**) Representative images and (**F**) densitometry analysis of formylglycinamide ribonucleotide amidotransferase (FGAMS) Western blot in control and SALS iAstrocytes. (**G**) Representative images and (**H**) densitometry analysis of phosphoribosylglycinamide formyltransferase/phosphoribosylglycinamide synthe-tase/phosphoribosylaminoimidazole synthetase (GART) Western blot in control and SALS iAstrocytes. (**I**) Representative images and (**J**) densitometry analysis of phosphoribosylaminoimidazole carboxylase/phosphoribosylaminoimidazole succinocarboxamide synthetase (PAICS) Western blot in control and SALS iAstrocytes. (**K**) Representative images and (**L**) densitometry analysis of HSP90 Western blot in control and SALS iAstrocytes. Data presented as mean and standard deviation of four biological replicates from three control and SALS iAstrocytes. Statistical analysis performed using Mann-Whitney test (B, F, H, J and L) or unpaired t test (D). *P ≤ 0.05. Where *P* value is not indicated results were non-significant. Uncropped Western images can be found in Supplementary Material.

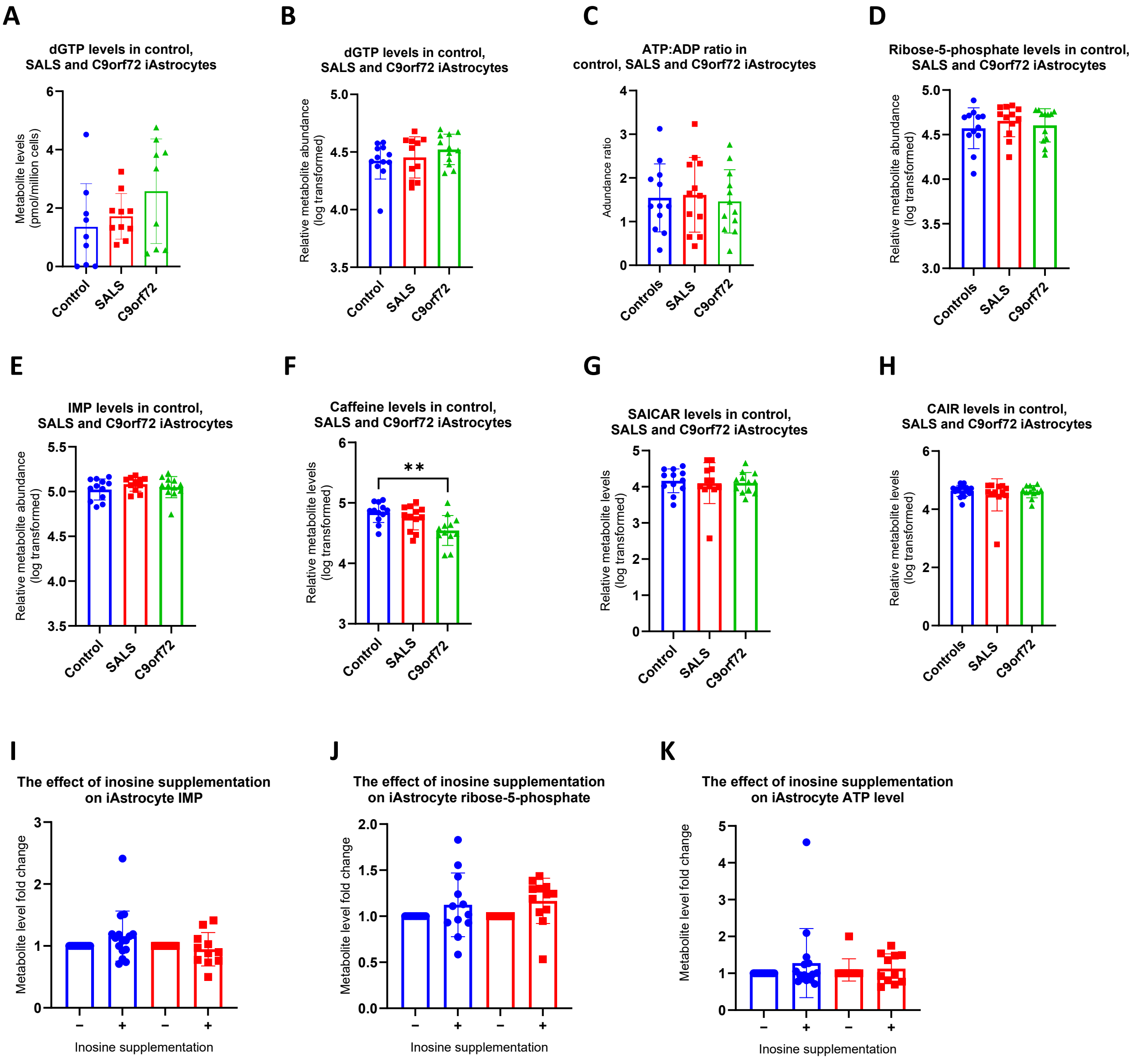

Supplementary Figure S2. Control, SALS and C9orf72 iAstrocyte (**A**) dGTP level as determined via coupled click chemistry and DNA polymerase-based assay. (**B**) dGTP level, (**C**) ATP:ADP ratio, (D) ribose-5-phosphate level, (**E**) IMP level, (**F**) caffeine level, (**G**) SAICAR level, and (H) CAIR level as measured via LC/MS. (**I**) IMP level, (**J**) ribose-5-phosphate level and (**K**) ATP level as measured via LC/MS, +/- 7.5mM inosine. iAstrocyte metabolite levels in the presence of inosine were normalised to metabolite levels in the absence of inosine by fold change analysis. Data presented as mean and standard deviation of four biological replicates from three control, three SALS and three C9orf72 iAstrocytes. Statistical analysis performed by unpaired t-tests (A-H), or Wilcoxon test (I, J, and K). **$P \leq 0.01$. Where P value is not indicated results were non-significant.

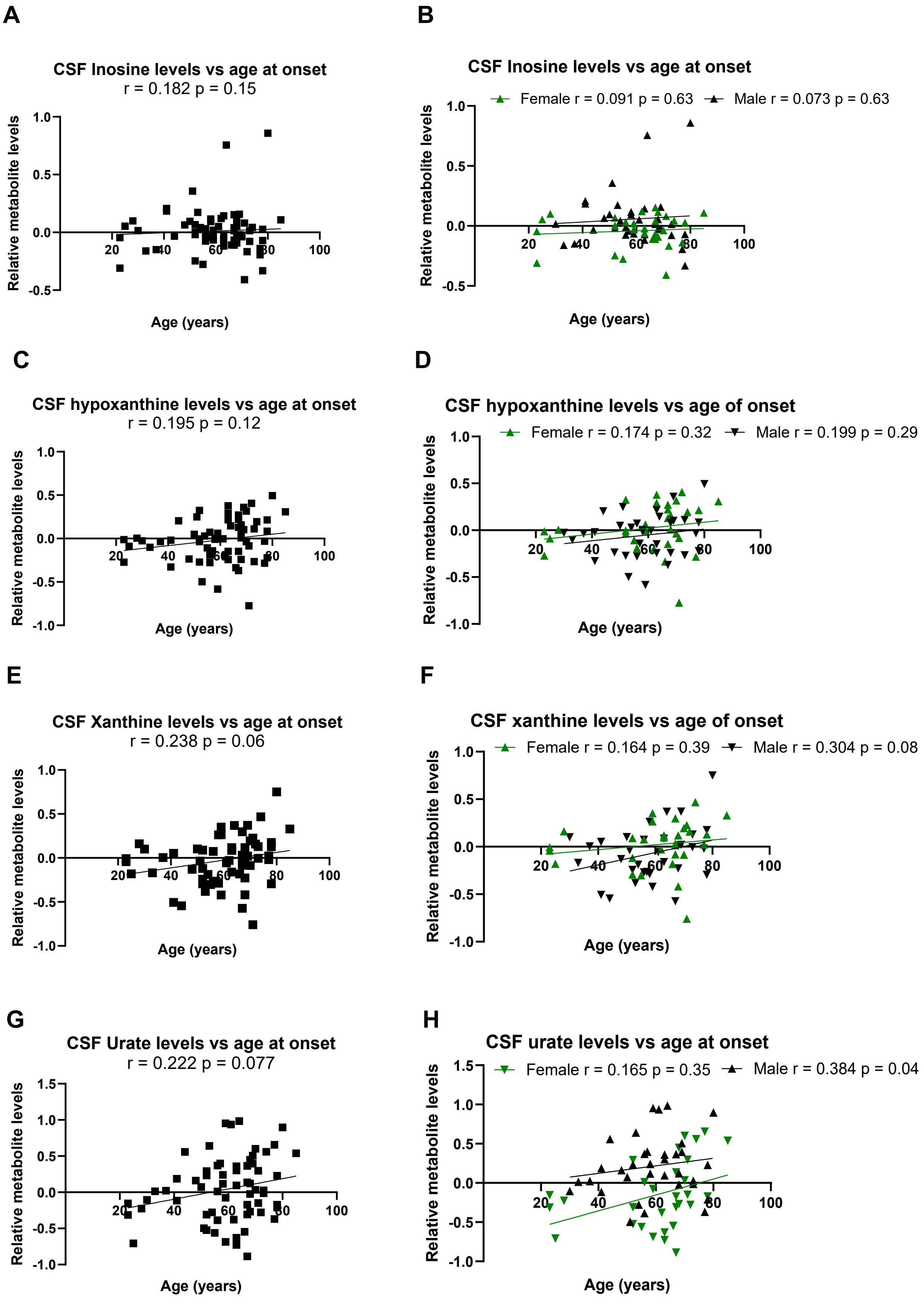

Supplementary Figure S3. CSF Purine levels are affected by age and sex in SALS Cases. (**A**) Age of onset vs CSF inosine levels, (**B**) Age of onset vs CSF inosine levels in male and females, (**C**) Age of onset vs CSF hypoxanthine levels, (**D**) Age of onset vs CSF hypoxanthine levels in males and females, (**E**) Age of onset vs CSF xanthine levels, (**F**) Age of onset vs CSF xanthine levels in males and females, (**G**) Age of onset vs CSF urate levels, (**H**) Age of onset vs CSF urate levels in males and females. Data presented for each individual SALS case, and statistical analysis performed by Pearsons or Spearman's correlation analysis.

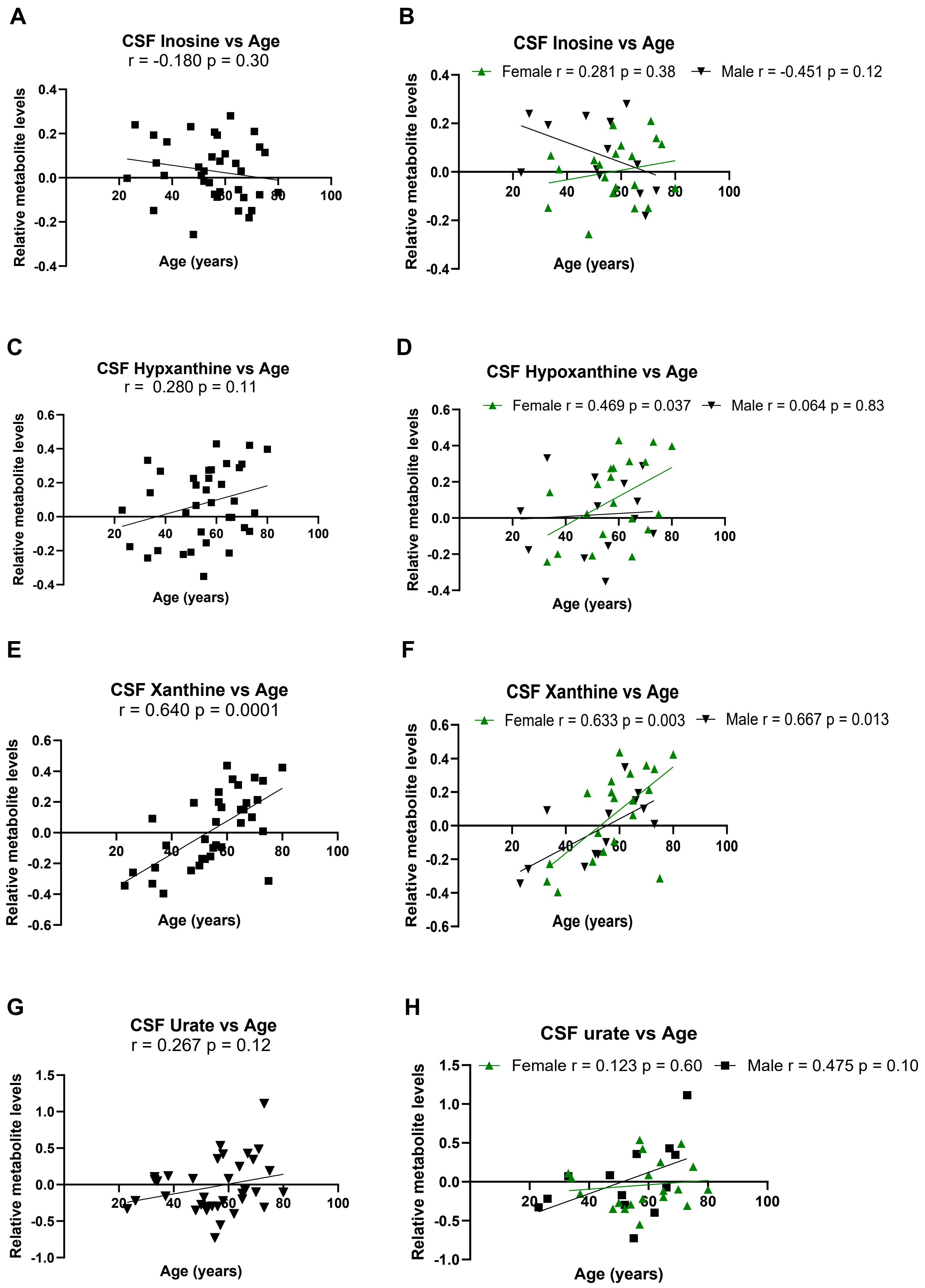

Supplementary Figure S4. CSF Purine levels are affected by age in control cases. (**A**) Age at collection vs CSF inosine levels, (**B**) Age at collection vs CSF inosine levels in male and females, (**C**) Age at collection vs CSF hypoxanthine levels, (**D**) Age at collection vs CSF hypoxanthine levels in males and females, (**E**) Age at collection vs CSF xanthine levels, (**F**) Age at collection vs CSF xanthine levels in males and females, (**G**) Age at collection vs CSF urate levels, (**H**) Age at collection vs CSF urate levels in males and females. Data presented for each individual SALS case, and statistical analysis performed by Pearsons or Spearman's correlation analysis.

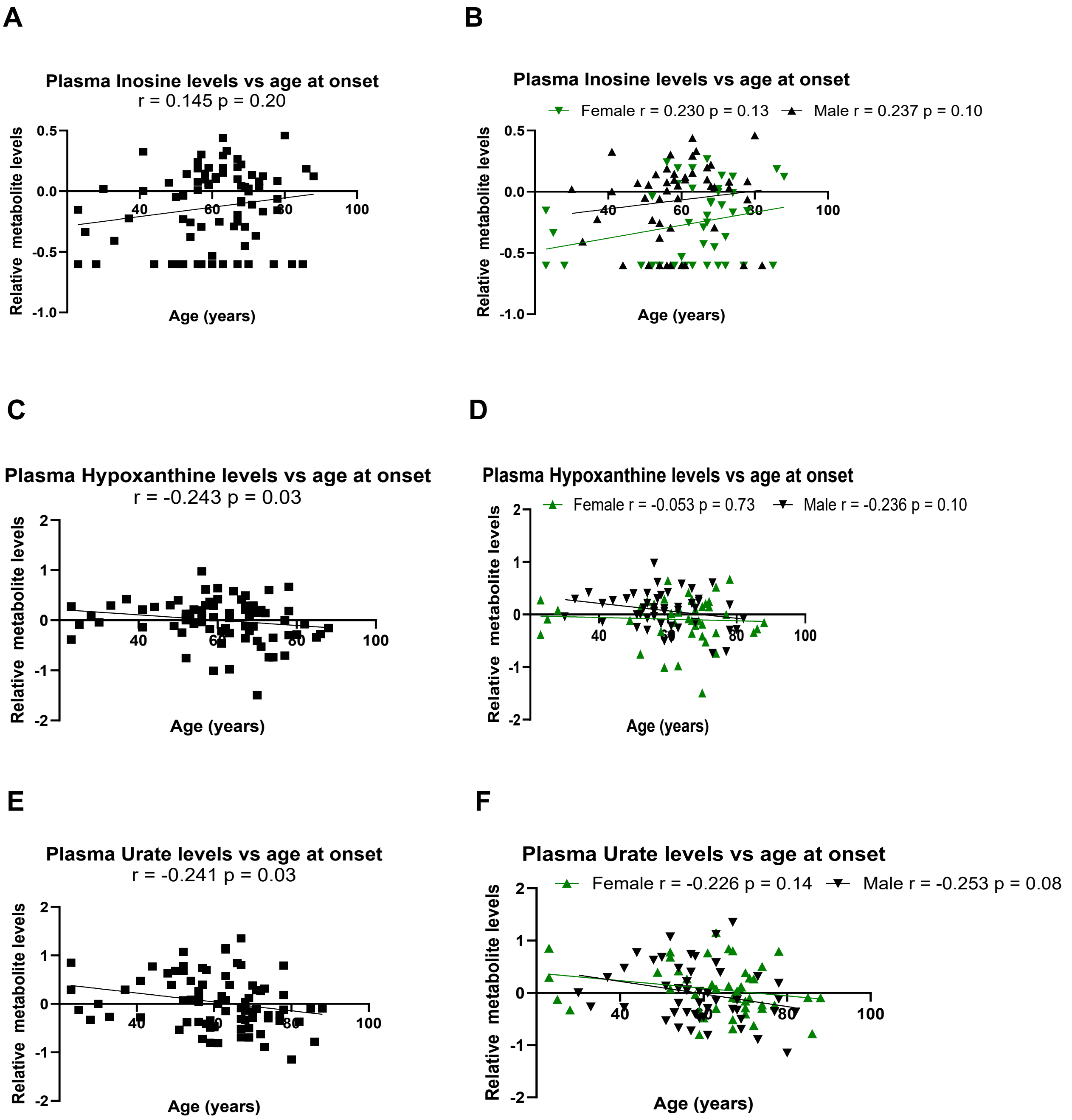

Supplementary Figure S5. Plasma purine levels vs age of onset in SALS cases (**A**) Age of onset vs plasma inosine levels, (**B**) Age of onset vs plasma inosine levels in male and females, (**C**) Age of onset vs plasma hypoxanthine levels, (**D**) Age of onset vs plasma hypoxanthine levels in males and females, (**E**) Age of onset vs plasma urate levels, (**F**) Age of onset vs plasma urate levels in males and females. Data presented for each individual SALS case, and statistical analysis performed by Pearsons or Spearman's correlation analysis.

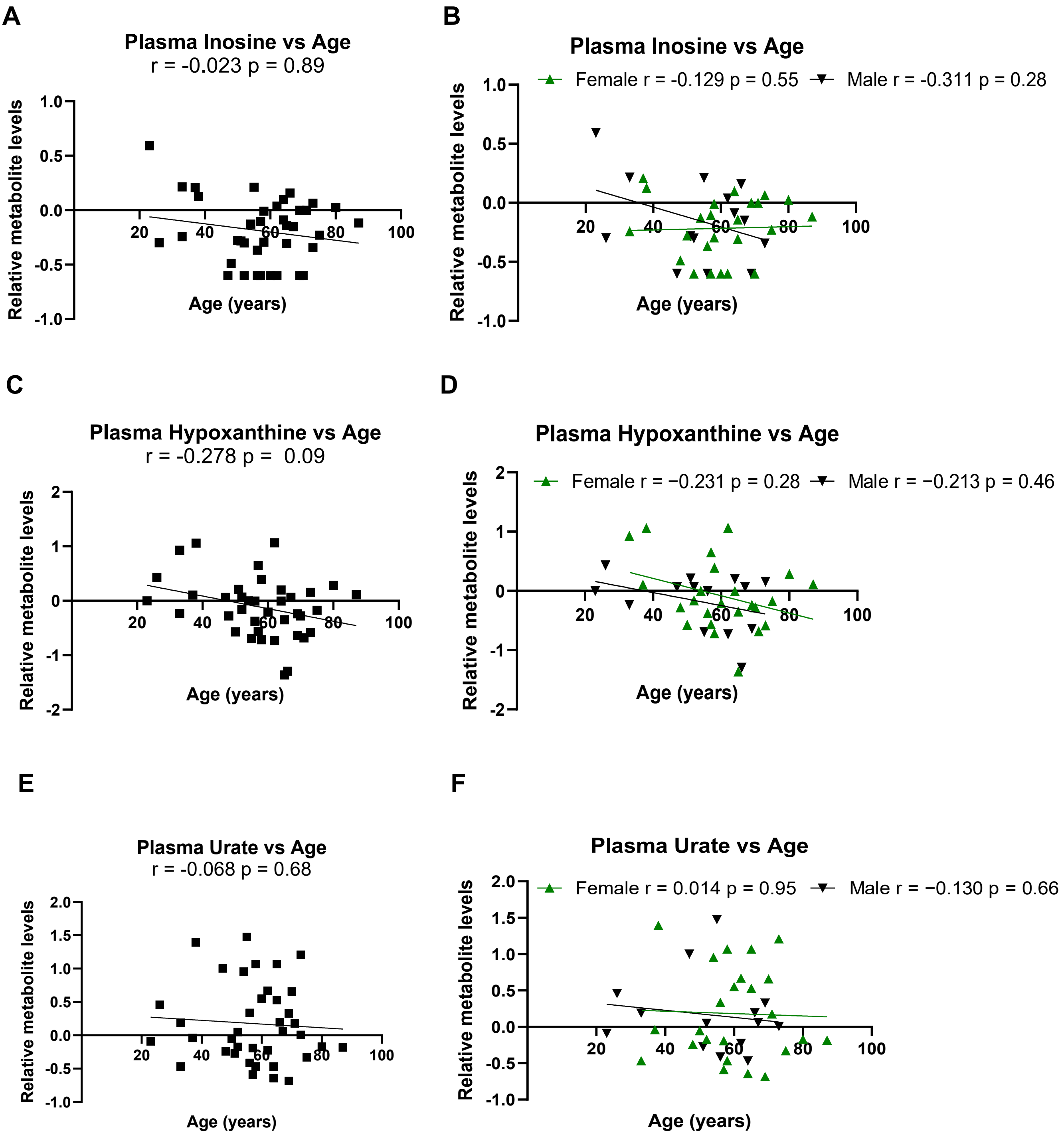

Supplementary Figure S6. Plasma purine levels are affected by age in control cases. (**A**) Age at collection vs plasma inosine levels, (**B**) Age at collection vs plasma inosine levels in male and females, (**C**) Age at collection vs plasma hypoxanthine levels, (**D**) Age at collection vs plasma hypoxanthine levels in males and females, (**E**) Age at collection vs plasma urate levels, (**F**) Age at collection vs plasma urate levels in males and females. Data presented for each individual SALS case, and statistical analysis performed by Pearsons or Spearman's correlation analysis.

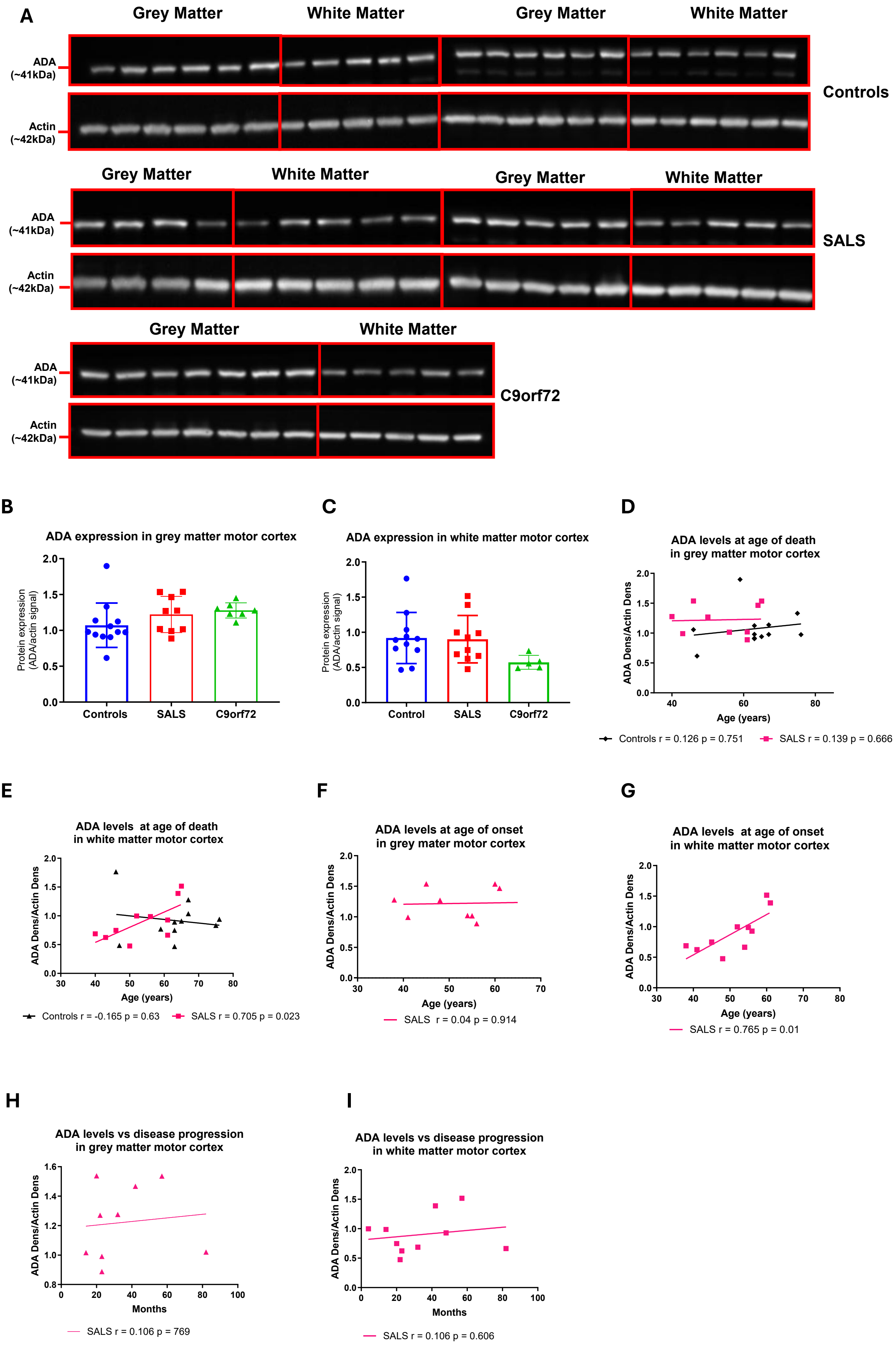

Supplementary Figure S7. ADA levels correlate with age of death in white matter cortex of SALS cases. (**A**,**B**) Grouped ADA levels in grey matter, (**A**,**C**) Grouped ADA levels in white matter, (**D**) Age of death vs grey matter ADA levels in SALS cases, (**E**) Age of death vs white matter ADA levels in SALS cases, (**F**) Age of onset vs grey matter ADA levels in SALS cases, (**G**) Age of onset vs white matter ADA levels in SALS cases, (**H**) Disease progression length vs grey matter ADA levels in SALS cases, (**I**) Disease progression length vs white matter ADA levels in SALS cases **(A**) Data presented as mean and standard deviation C9orf72 data from Hall et al is added as a graphical comparison. **(E-J**) Data presented for each individual SALS case, and statistical analysis performed by Pearsons or Spearman's correlation analysis. Where p value is not indicated results were non-significant. Includes data and representative blots for C9orf72 and Controls used in Hall et al 2016 [14].

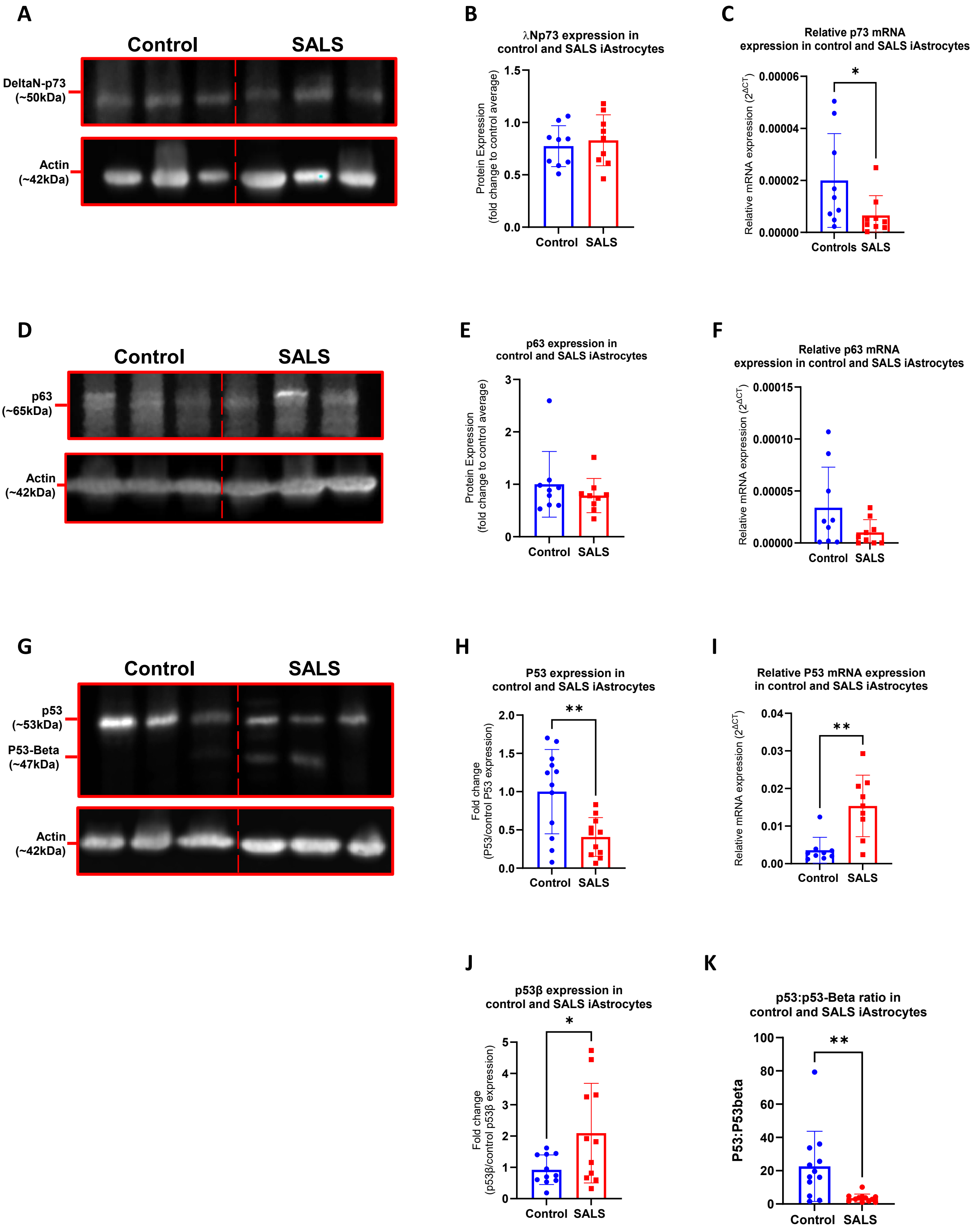

Supplementary Figure S8. ADA transcription factor expression in control and SALS iAstrocytes. (**A**) Delta-Np73 Western blot representative images. (**B**) Delta-Np73 densitometry analysis. (**C**) Delta-Np73 relative mRNA expression. (**D**) p63 Western blot representative images. (**E**) p63 densitometry analysis. (F) p63 relative mRNA expression. (G) p53 Western blot representative images. (**H**) p53 densitometry analysis. (**I**) p53 relative mRNA expression. (**J**) p53β densitometry analysis. **(K)** p53: p53β ratio densitometry analysis. Data presented as mean and standard deviation of 3/4 biological replicates from three control and three SALS iAstrocytes. Statistical analysis by unpaired t-test or Mann Whitney test. $*P \leq 0.05$, $**P \leq 0.01$, $***P \leq 0.001$. Where P value is not indicated results were non-significant. Uncropped Western images can be found in Supplementary Material.

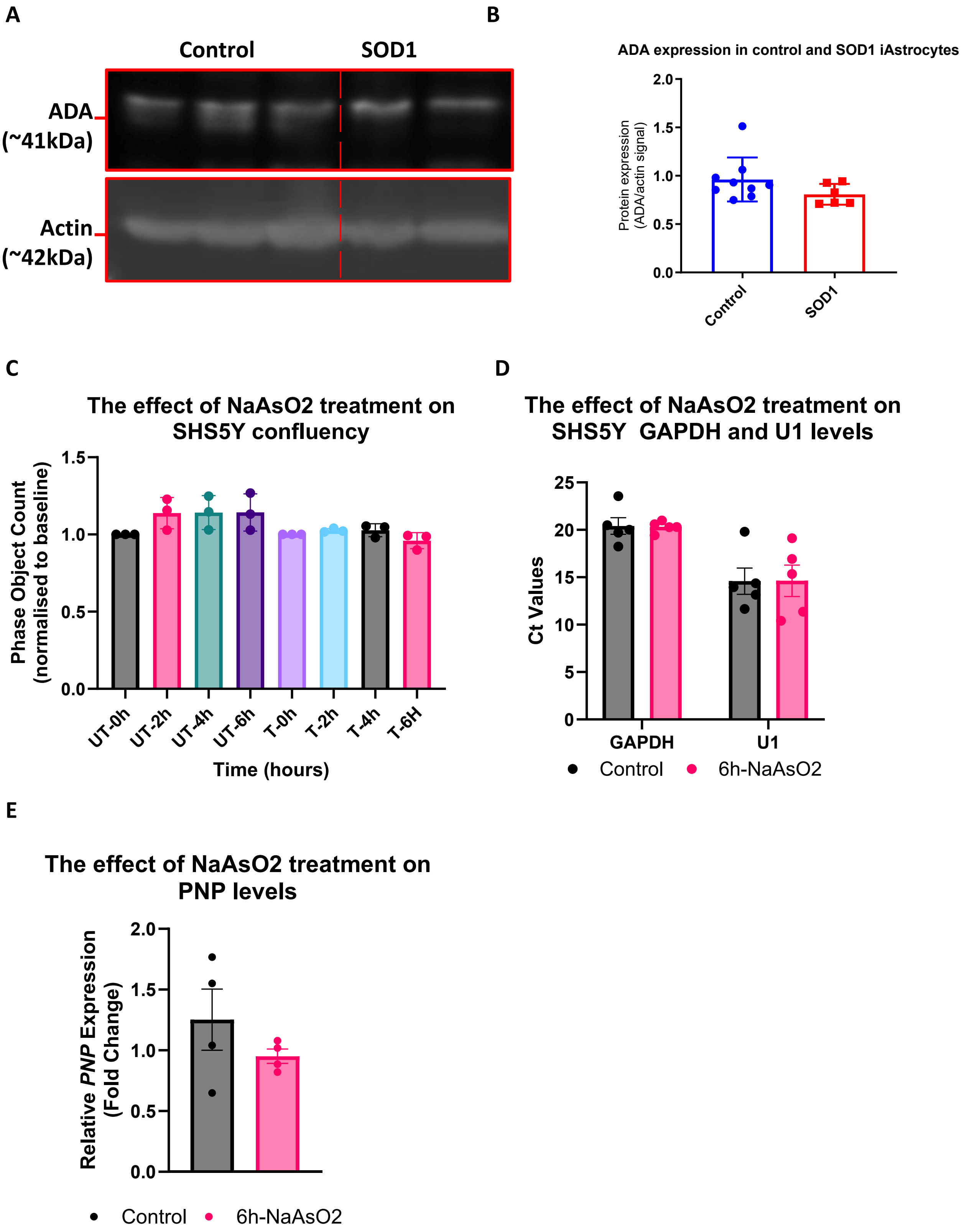

Supplementary Figure S9. ADA expression is unaltered in SOD1 iAstrocytes. (**A**) Representative images and (**B**) densitometry analysis of ADA Western blot in control and SOD1 iAstrocytes. (**C**) The effect of Sodium Arsenite (NaAsO2) on SHS5Y confluency up to six hours). (**D**) The effect of Sodium Arsenite (NaAsO2) on SHS5Y GAPDH/U1 levels. (**E**) The effect of Sodium Arsenite (NaAsO2) on SHS5Y PNP levels. Data presented as mean and standard deviation of three-five biological replicates from three control and two SOD1 iAstrocytes (A-B). Statistical analysis performed using Mann-Whitney test (B/D). Kruskal Wallis with Dunn's post test (C). Where *P* value is not indicated results were non-significant. Uncropped Western images can be found in Supplementary Material.

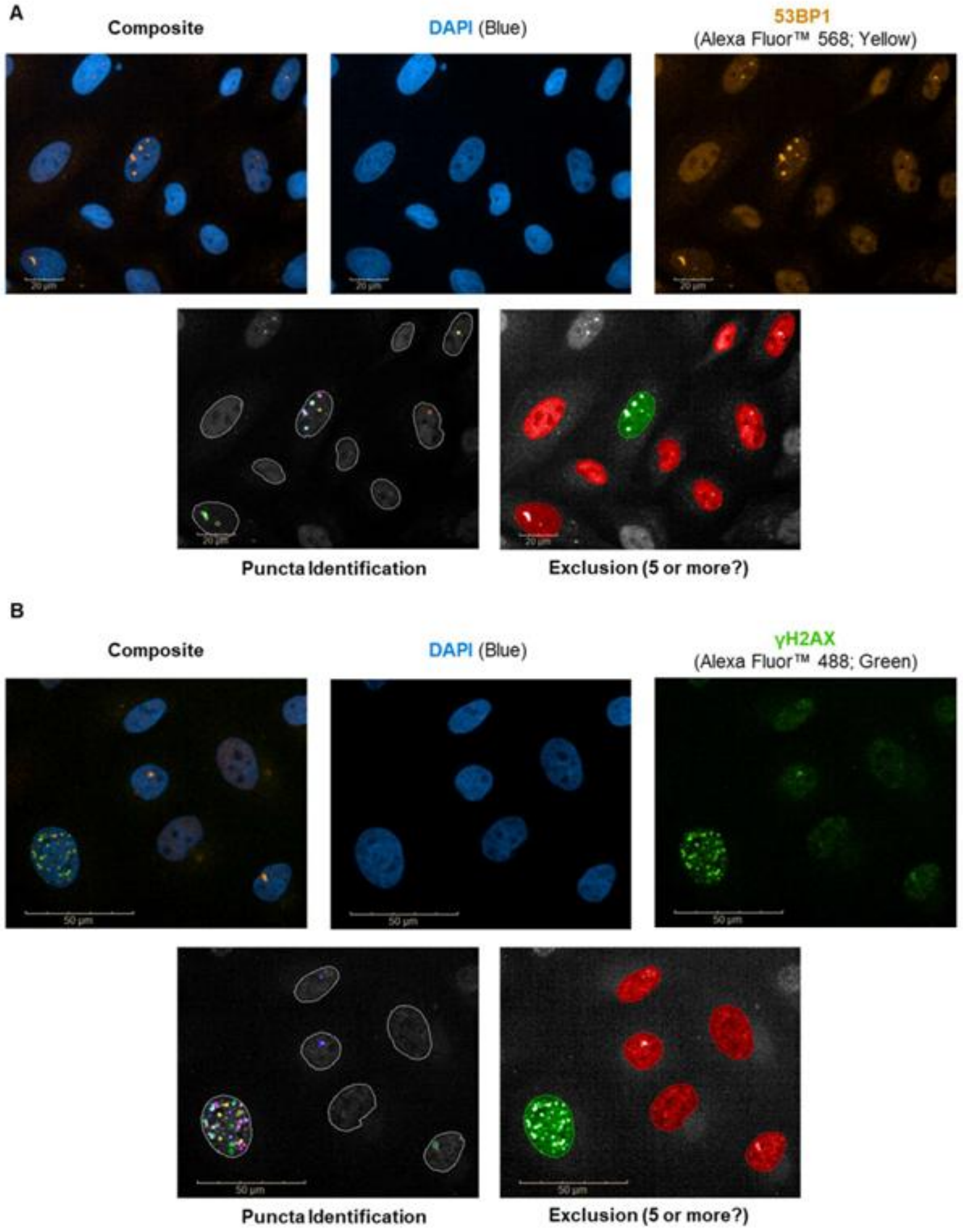

Supplementary Figure S10. Identification of cells with ≥5 Puncta for DNA Damage Quantification. (**A**) Identification of cells with 5 or more 53BP1 nuclear puncta. (**B**) Identification of cells with 5 or more γH2AX nuclear puncta. Performed using a custom protocol using Harmony 5.2 (Revvity) Software.

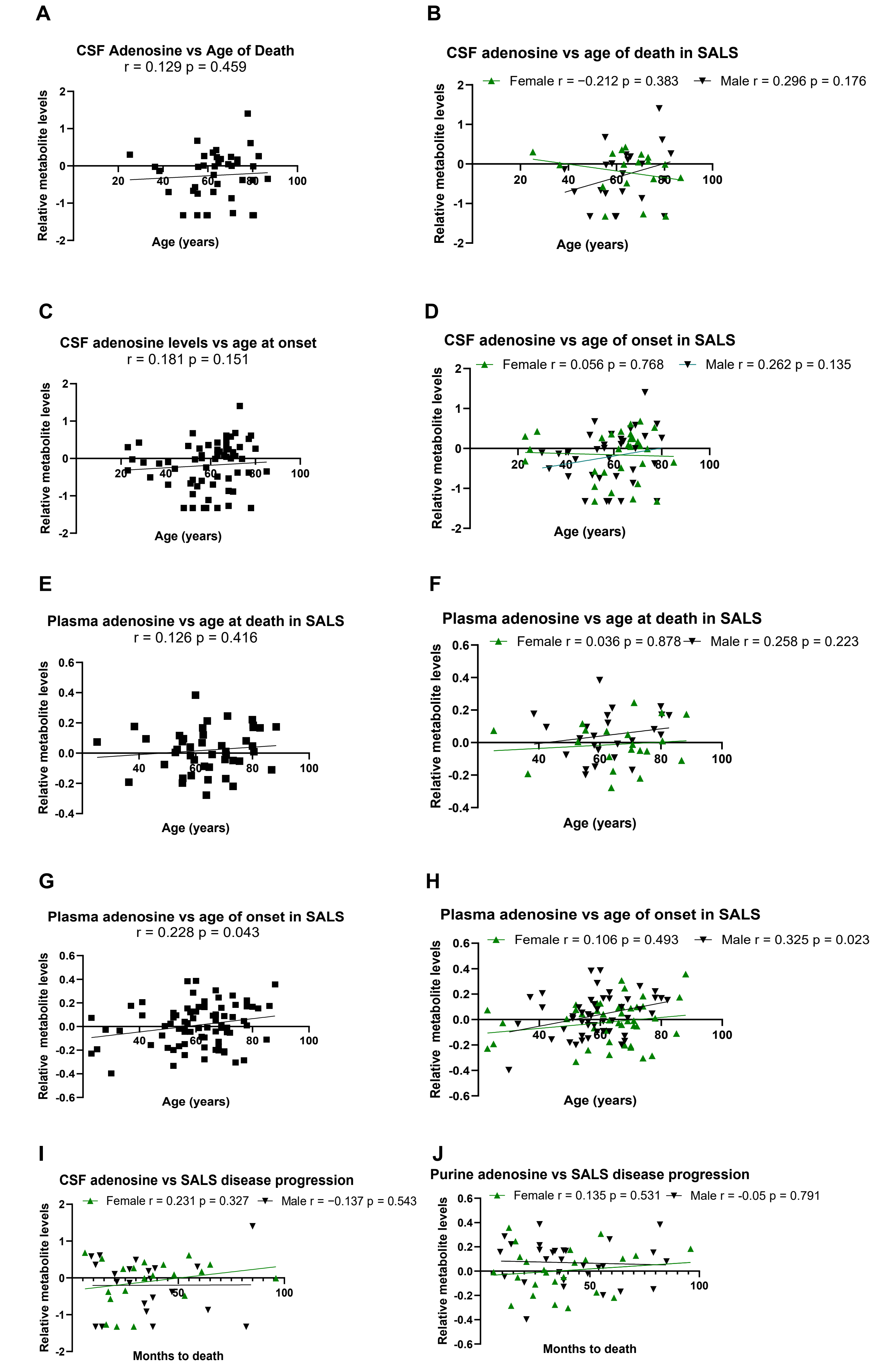

Supplementary Figure S11. CSF and plasma adenosine levels in SALS cases (**A**) Age of death vs CSF adenosine levels, (**B**) Age of death vs CSF adenosine levels in male and females, **(C)** Age of onset vs CSF adenosine levels, (**D**) Age of onset vs CSF adenosine levels in male and females, (**E**) Age of death vs plasma adenosine levels, (**F**) Age of death vs plasma adenosine levels in males and females, (**G**) Age of onset vs plasma adenosine levels, (**H**) Age of onset vs plasma adenosine levels in males and females, (**I**) Disease progression vs CSF adenosine levels in males and females, (**J**) Disease progression vs plasma adenosine levels in males and females. Data presented for each individual SALS case, and statistical analysis performed by Pearsons or Spearman's correlation analysis.

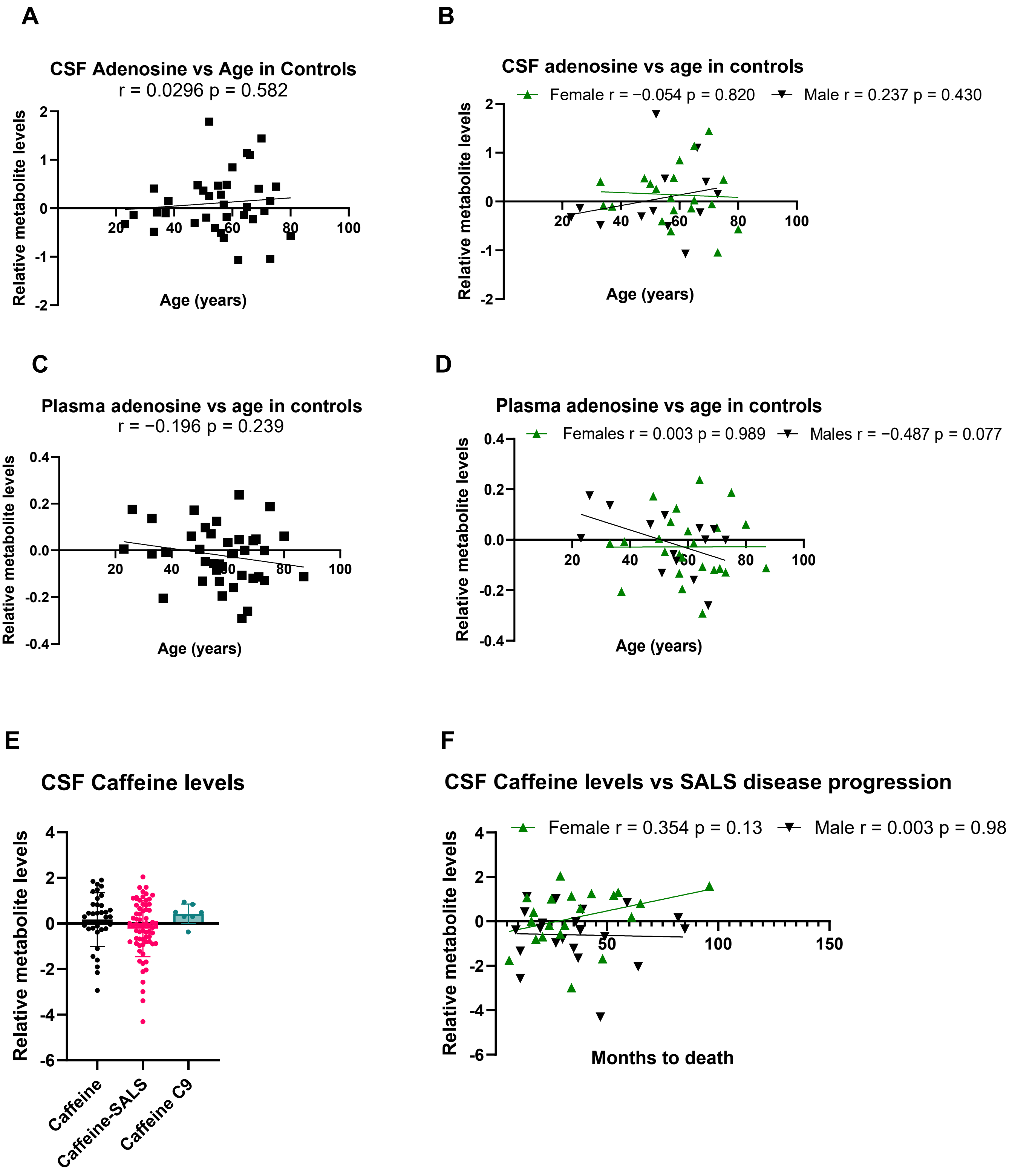

Supplementary Figure S12. CSF and plasma adenosine levels in controls . (**A**) Age vs CSF adenosine levels, (**B**) Age vs CSF adenosine levels in male and females, (**C**) Age vs plasma adenosine levels, (**D**) Age vs plasma adenosine levels in males and females. (**E**) CSF caffeine levels. (**F**) Disease progression vs CSF caffeine levels. Data presented for each individual control case, and statistical analysis performed by Pearsons or Spearman's correlation analysis or Mann Whiteny analysis (E).
