# Supplementary material for: Adenosine Deaminase-Mediated Purine Dysfunction Leads to DNA Repair Inhibition and Senescence in Sporadic Amyotrophic Lateral Sclerosis
Source: Int J Mol Sci. 2026 Sep 16;27(18):8236. doi: 10.3390/ijms27188236 (PMC13607153; doi:10.3390/ijms27188236)
Supplement: Supplementary file 1 [file ijms-27-08236-s001.zip › Supplementary Materials and Methods FINAL-2 080926.pdf]

Cell line	Sex (M/F)	Age at biopsy (years)	Disease duration (months)
Con-155	M	40	N/A
Con-209	F	69	N/A
Con-3050	M	65	N/A
SALS-9	F	61	21
SALS-12	M	47	72
SALS-17	M	29	90
C9-78	M	66	31.7
C9-183	M	50	27
C9-201	F	66	19.4
SOD1-100	F	63	100
SOD1-102	F	40	200

Table S1. Details of control, SALS, C9orf72 and SOD1 skin biopsy donors.

Subject	Sex (M/F)	Age-at-onset (years)	Age-at-death (years)	White/Grey matter/Both	Disease duration (months)
Con-129/94	M	N/A	63	Both	N/A
Con-025/95	M	N/A	67	Both	N/A
Con-178/95	F	N/A	63	Both	N/A
Con-293/91	F	N/A	65	Both	N/A
Con-072/92	F	N/A	76	Both	N/A
Con- 109/95	M	N/A	46	Both	N/A
Con-85/07	F	N/A	59	Both	N/A
Con-147/95	M	N/A	47	Both	N/A
Con-05/07	M	N/A	63	Both	N/A
Con-098/07	M	N/A	67	Both	N/A
Con-10/96	F	N/A	63	Grey	N/A
Con-071/92	M	N/A	75	Both	N/A

Table S2. Details of control white and grey matter donors

Subject	Sex (M/F)	Age-at-onset (years)	Age-at-death (years)	White/Grey matter/Both	Disease duration (months)
SALS-96/159	M	41	43	Both	23
SALS-069/06	M	48	50	Both	22
SALS-212/90	M	55	56	Both	14
SALS-075/08	F	54	61	Both	82
SALS-039/05	F	56	61	Both	48
SALS-42/05	M	60	65	Both	57
SALS-005/10	M	38	40	Both	32
SALS-148/90	F	45	46	Both	20
SALS-49/05	F	61	64	Both	42
SALS-150/93	F	52	52	White	4

Table S3. Details of SALS white and grey matter donors.

Antibody	Species	Western blot dilution	Immunofluorescence dilution	Source	Cat. No.
53BP1	Rabbit	1:2000	1:1000	Novus Biologicals	NB-100-304
ADA	Mouse	1:250-1,000	N/A	Santa Cruz	sc28346
ADK	Rabbit	1:1000	N/A	Proteintech	66929-1-IG
ADSL	Rabbit	1:4000	N/A	Proteintech	15264-1-AP
Actin	Rabbit	1:3000	N/A	Proteintech	20536-1-AP
ATIC	Rabbit	1:4000	N/A	Proteintech	10726-1-AP
CD73	Rabbit	1:2000	N/A	Proteintech	12231-1-AP
FGAMS	Rabbit	1:7500	N/A	ThermoFisher	A304-219A
GART	Rabbit	1:2000	N/A	Proteintech	13659-1-AP
HGPRT	Rabbit	1:2000	N/A	Proteintech	15059-1-AP
HSP90	Rabbit	1:5000	N/A	Proteintech	60318-1-lg
p16	Mouse	1:2000	1:500	Invitrogen	MA5-17054
p21	Rabbit	1:2000	1:500	Proteintech	10355-1-AP
p53	Mouse	N/A	1:4000	Santa-Cruz	sc-126
p73	Mouse	1:2000	N/A	ThermoFisher	38C674.2
PAICS	Rabbit	1:3000	N/A	Proteintech	12967-1-AP
PNP	Mouse	1:1000	N/A	R&D Systems	MAB6486
TDP-43	Rabbit	1:1000	N/A	Proteintech	12892-1-AP
Vimentin	Chicken	N/A	1:1000	Millipore	ab5733
XO	Rabbit	1:2000	N/A	Proteintech	55156-1-AP
γH2AX	Mouse	1:2000	1:500	Abcam	ab26350

Table S4. Details of the primary antibodies used in this study.

Species	Western blot dilution	Immunofluorescence dilution	Excitation/Emission (nm)	Source	Cat. No.
Hoescht	N/A	1:2,000-10,000	350/461	ThermoFisher	H3570
Anti-Mouse	1:5,000	N/A	N/A	Abcam	ab97040
	1:2500	N/A	N/A	Cell Signalling	7076
Anti-Rabbit	1:5,000	N/A	N/A	Sigma-Aldrich	A0545
Fluorescent Anti-Mouse	N/A	1:1000	499/520	Invitrogen	A11001
Fluorescent Anti-Rabbit	N/A	1:1000	579/603	Invitrogen	A11011
Fluorescent Anti-Chicken	N/A	1:1000	650/671	Invitrogen	A78952

Table S5. Details of the secondary antibodies used in this study.

Primer	Fwd/Rev (F/R)	Sequence (5'-3')
ADA	F	CTGCTGAACGTCATTGGCATGG
ADA	R	GGCGATCCTTTTGATAGCCTCC
ADA Drosophila	F	GGCATCAAAAGCCTATGTGATCT
ADA Drosophila	R	CGCGCACAACTTCAGGAAA
CD73	F	CTCCTCTCAATCATGCCGCT
CD73	R	TGGATTCCATTGTTGCGTTCA
HGPRT	F	CATTATGCTGAGGATTTGGAAAGG
HGPRT	R	CTTGAGCACACAGAGGGCTACA
Tub8	F	TGGGCCCGTCTGGACCACAA
Tub8	R	TCGCCGTCACCGGAGTCCAT
U1	F	CCATGATCACGAAGGTGGTT
U1	R	ATGCAGTCGAGTTTCCCACA
p53	F	CCTCAGCATCTTATCCGAGTGG
p53	R	TGGATGGTGGTACAGTCAGAGC
p63	F	CAGGAAGACAGAGTGTGCTGGT
p63	R	AATTGGACGGCGGTTCATCCCT
p73	F	CATGGAGACGGAGGACACGTACT
p73	R	TGCCGATAGGAGTCCACCAGTG
GAPDH	F	TCGCCAGCCGAGCCA
GAPDH	R	GAGTTAAAAGCAGCCCTGGTG
PNP	F	TTTCCCCGAAGTACAGTGCC
PNP	F	CCCTCACTGGGAATGTCACC
RPL32 Drosophila	F	GCTAAGCTGTCGCACAAATGG
RPL32 Drosophila	R	CGGCGACGCACTCTGTT

Table S6. Details of the primer pairs used in this study including 5'-3' sequence.
